# Quorum sensing and stress-activated MAPK signaling repress yeast to hypha transition in the fission yeast *Schizosaccharomyces japonicus*

**DOI:** 10.1371/journal.pgen.1008192

**Published:** 2019-05-31

**Authors:** Elisa Gómez-Gil, Alejandro Franco, Marisa Madrid, Beatriz Vázquez-Marín, Mariano Gacto, Jesualdo Fernández-Breis, Jero Vicente-Soler, Teresa Soto, José Cansado

**Affiliations:** 1 Yeast Physiology Group, Departmento de Genética y Microbiología, Facultad de Biología. Universidad de Murcia, Murcia, Spain; 2 Departamento de Informática y Sistemas, Facultad de Informática. Universidad de Murcia, Murcia, Spain; University College Dublin, IRELAND

## Abstract

Quorum sensing (QS), a mechanism of microbial communication dependent on cell density, governs developmental decisions in many bacteria and in some pathogenic and non-pathogenic fungi including yeasts. In these simple eukaryotes this response is mediated by the release into the growth medium of quorum-sensing molecules (QSMs) whose concentration increases proportionally to the population density. To date the occurrence of QS is restricted to a few yeast species. We show that a QS mediated by the aromatic alcohols phenylethanol and tryptophol represses the dimorphic yeast to hypha differentiation in the fission yeast *S*. *japonicus* in response to an increased population density. In addition, the stress activated MAPK pathway (SAPK), which controls cell cycle progression and adaptation to environmental changes in this organism, constitutively represses yeast to hypha differentiation both at transcriptional and post-translational levels. Moreover, deletion of its main effectors Sty1 MAPK and Atf1 transcription factor partially suppressed the QS-dependent block of hyphal development under inducing conditions. RNAseq analysis showed that the expression of *nrg1*^*+*^, which encodes a putative ortholog of the transcription factor Nrg1 that represses yeast to hypha dimorphism in *C*. *albicans*, is downregulated both by QS and the SAPK pathway. Remarkably, Nrg1 may act in *S*. *japonicus* as an activator of hyphal differentiation instead of being a repressor. *S*. *japonicus* emerges as an attractive and amenable model organism to explore the QS mechanisms that regulate cellular differentiation in fungi.

## Author summary

Quorum sensing is a relevant mechanism of communication dependent on population density that controls cell development and pathogenesis in microorganisms including fungi. We describe a quorum sensing mediated by the release of aromatic alcohols in the growth medium that blocks hyphal development in the fission yeast *Schizosaccharomyces japonicus*. This is the first description of such a mechanism in the fission yeast lineage, and confirms its expansion along Ascomycota fungi. The stress-responsive pathway (SAPK), which regulates fungal growth and differentiation, limits hyphal growth in *S*. *japonicus* in a constitutive fashion, and nonfunctional SAPK mutants are partially insensitive to quorum sensing and able to form hyphae in high cell density cultures. Nrg1, an important factor that blocks hyphal development in the pathogen *Candida albicans*, activates hyphal growth in *S*. *japonicus*, and its expression is counteracted by both quorum sensing and the SAPK pathway. Nrg1 function may thus have diverged evolutionary in this organism from being a repressor to an activator of hyphal development. *S*. *japonicus* emerges as a suitable model organism to explore the intricate mechanisms regulating fungal differentiation.

## Introduction

The highly conserved mitogen-activated protein kinase (MAPK) signaling pathways are key players in eukaryotic cells to elicit proper adaptive responses to environmental changes. Once activated in response to external and internal cues, MAPKs phosphorylate a wide range of extranuclear proteins and/or shift into the nucleus to phosphorylate transcription factors that do, in turn, execute transcriptional programs that promote cellular adaptation to the triggering stimulus [1]. The stress-activated pathway (SAPK), one of the three MAPK pathways present in the rod-shaped fission yeast *Schizosaccharomyces pombe*, shows significant functional homology to the mammalian p38 pathway [2], and plays a critical role in the control of cell cycle and the general response to stress. Its central element, the MAPK Sty1, becomes phosphorylated at two conserved threonine and tyrosine residues within its activation loop in response to multiple stressful conditions [2]. Activated Sty1 then moves in turn to the nucleus and phosphorylates the bZIP domain transcription factor Atf1 to modulate the expression of a group of genes including the CESR (Core Environmental Stress Response) genes, which participate in the consequent adaptive cell response. Activated Sty1 also controls mRNA stabilization, cell cycle progression at the G2/M transition, and polarized growth during growth and stress [2, 3].

The fission yeast species *S*. *japonicus* is becoming an attractive model organism to explore evolutionary physiological and developmental changes within the *Schizosaccharomyces* clade [4, 5]. However, the biological significance of the stress-activated MAP kinase signaling pathway in *S*. *japonicus* remains to be established. Both *S*. *pombe* and *S*. *japonicus* grow by binary fission during vegetative growth and share similar mechanisms for conjugation and sporulation. However, *S*. *japonicus* has distinctive features including a defective respiration, a highly dynamic actin cytoskeleton, and semi-open mitosis [6–12]. *S*. *pombe* and *S*. *japonicus* are able to show pseudohyphal/hyphal growth under specific conditions, although the penetrance of such phenotype is very different in the two species. Pseudohyphal growth in *S*. *pombe* requires high cell density and occurs in strains of specific genetic backgrounds growing in media with low nitrogen content and abundant carbon source [13–15]. In contrast, *S*. *japonicus* cells undergo robust yeast to hypha differentiation in either liquid or solid media under various conditions including nutrient stress [16–19], or DNA damage induced with camptothecin (CPT), a topoisomerase I inhibitor [19, 20]. Indeed, low CPT concentrations trigger yeast to hypha transition through a mechanism involving the Chk1 kinase and the Rad3–Rad9 pathway, but without induction of checkpoint arrest [20, 21]. Moreover, differentiation is a reversible process, and hyphal cells quickly return to the fission yeast morphology after drug removal from the growth medium [20]. This feature makes *S*. *japonicus* a suitable model organism to unveil novel and crucial mechanisms for the comprehension of the dimorphism process.

Quorum sensing (QS) is a mechanism of microbial communication dependent on cell density that governs developmental decisions in many bacterial species and in some pathogenic and non-pathogenic yeast species like *Candida albicans* and *Saccharomyces cerevisiae* [22–24]. The main effectors of QS, known as auto-inducers or quorum-sensing molecules (QSM), are secreted into the growth medium and their concentration increases proportionally to the population density [22, 23]. Fungal QSMs, which include acyclic and/or aromatic alcohols such as farnesol, tyrosol, phenylethanol and tryptophol, impact morphogenesis, germination of macroconidia and apoptosis [22, 23]. Consequently, they also play a role in fungal pathogenesis, as is the case with farnesol and tyrosol, which modulate yeast to hyphae morphological switch and biofilm formation in *C*. *albicans* [22, 23].

In this work we demonstrate the existence of an inducible cell density dependent QS in *S*. *japonicus* that negatively controls yeast to hypha transition and that is mediated by aromatic alcohols. This mechanism is reinforced by the SAPK pathway, which also negatively regulates hyphal initiation and maintenance in a constitutive fashion. Remarkably, QS and the Sty1-Atf1 pathway downregulate the expression of Nrg1, which, contrary to its *C*. *albicans* ortholog [25, 26], is not a repressor but an activator of hyphal differentiation.

## Results

### A quorum sensing mechanism mediated by aromatic alcohols inhibits yeast to hypha transition in *S*. *japonicus*

**Fig 1:**
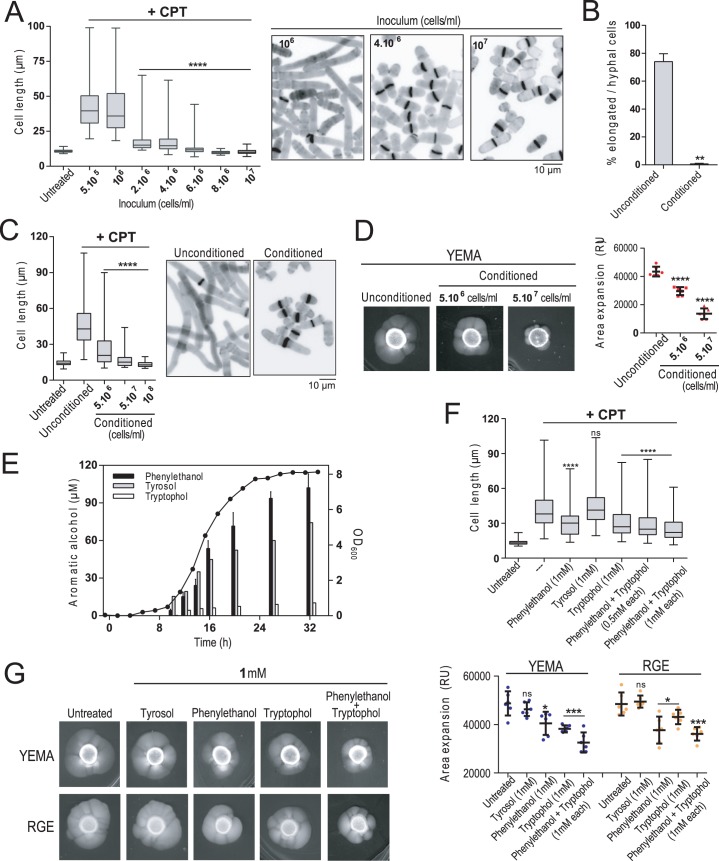
Quorum sensing negatively regulates yeast to hypha transition in *S*. *japonicus*. (A) Exponentially growing *S*. *japonicus* wild type cells were recovered by filtration, inoculated at the indicated initial cell densities in high glucose (6%) YES medium, and incubated for 6h in the presence or absence of 0.2 μM CPT. Cell length is represented as box and whisker plots. Data obtained after quantification of one experiment performed per triplicate (n≥ 200 cells/strain) is shown. ****, *P*<0.0001, as calculated by unpaired Student´s *t* test. Fixed cells from representative experiments were stained with calcofluor white and observed by fluorescence microscopy. Scale bar: 10 μm. (B) *S*. *japonicus* wild type cells were inoculated at low density (10^6^ cells/ml) in fresh YES medium (3% glucose) or in filter-sterilized conditioned medium (~3% remaining glucose concentration) obtained from a culture of the same strain growing to high cell density (≥10^8^cells/ml), and supplemented with 0.2 μM CPT. Aliquots were recovered after 6h of incubation, stained with calcofluor white, and the percentage of elongated/hyphal cells was determined by fluorescence microscopy. Data are expressed as mean ± SD and correspond to biological triplicates (n≥200 cells/sample). **, *P*<0.005, as calculated by unpaired Student´s *t* test. (C) Cell length represented as box and whisker plots in *S*. *japonicus* wild type cells growing exponentially for 6h in the presence of 0.2 μM CPT in either fresh (unconditioned) or conditioned YES medium (3% glucose) obtained from a culture of the same strain growing at a density of 5.10^6^, 5.10^7^ or 10^8^ cells/ml. The experiment was performed per triplicate (n≥ 200 cells) and quantification of one is shown. ****, *P*<0.0001, as calculated by unpaired Student´s *t* test. CPT-treated cells growing in unconditioned and conditioned medium (density of 5.10^7^ cells/ml) stained with calcofluor white and observed by fluorescence microscopy. Scale bar: 10 μm. (D) Cells from *S*. *japonicus* wild type cells growing in YES medium (2.10^6^) were spotted on YEMA plates prepared with unconditioned or conditioned medium obtained from a culture of the same strain growing to a density of 5.10^6^ or 5.10^7^ cells/ml, incubated at 30°C for 7 days, and then photographed. The total area of mycelial expansion (expressed as relative units) was measured (n>6) and is represented as scatter plot. ****, *P*<0.0001, as calculated by unpaired Student´s *t* test. (E) Growth curve of wild type *S*. *japonicus* in high glucose (6%) YES medium was followed by determining OD_600_ values at different times (black circles). Media supernatants were recovered by filter-sterilization at the indicated time points, and the concentration of phenylethanol (black bars), tyrosol (grey bars), and tryptophol (white bars) secreted into the growth medium was determined by GC/MS or HPLC/MS analysis. Data are expressed as mean ± SD and correspond to biological triplicates. (F) Exponentially growing *S*. *japonicus* wild type cells were inoculated at an initial cell density of 10^6^ cells/ml in high glucose (6%) YES medium and incubated for 6h with 0.2 μM CPT without further treatment (untreated) or in the presence of the indicated amounts of either phenylethanol, tyrosol, tryptophol, or phenylethanol plus tryptophol. Cell length is represented as box and whisker plots. Data obtained after quantification of one experiment performed per triplicate (n≥ 200 cells/sample) is shown. ****, *P*<0.0001; ns, not significant, as calculated by unpaired Student´s *t* test. (G) Cells from *S*. *japonicus* wild type cells growing in YES medium (2.10^6^) were spotted on YEMA or RGE plates in the absence or presence of the indicated amounts of either phenylethanol, tyrosol, tryptophol, or phenylethanol plus tryptophol, incubated at 30°C for 7 days, and then photographed. The total area of mycelial expansion (expressed as relative units) was measured (n≥6) and is represented as scatter plot. *, *P*<0.05; ***, *P*<0.001; ns, not significant, as calculated by unpaired Student´s *t* test.

Treatment of exponentially growing wild type *S*. *japonicus* yeast cells with very low doses of CPT (0.2 μM) prompts their differentiation into filamentous cells (hyphae) with relative quick kinetics and without inducing a checkpoint arrest [20]. We noticed that the ability of *S*. *japonicus* yeast cells to differentiate into filaments and/or hyphae inversely correlates with the initial population density present in the medium. In our experimental setup the average cell length of filamentous wild type cells after 6h of incubation in the presence of 0.2 μM CPT (~40 μm) did not changed significantly when the initial inoculum was of 5.5.10^5^ or 10^6^ cells/ml (Fig 1A). However, cell differentiation became strongly limited when cells where inoculated at ≥ 2.10^6^ cells/ml (~18 μm), and progressively reduced as cell density was raised to 10^7^ cells/ml (~10 μm) (Fig 1A). *S*. *japonicus* cells divided normally during the course of the experiment at these population densities (Fig 1A), confirming that inhibition of the dimorphic switch was not due to a growth arrest. Therefore, a quorum sensing mechanism might be responsible for the inhibition of hyphal differentiation of *S*. *japonicus* at high cell densities. As support for this hypothesis, the elongated/hyphal cells induced with CPT where almost undetectable when the wild type strain was inoculated at a low density (10^6^ cells/ml) in filter-sterilized conditioned medium obtained from a high cell density culture of the same strain (>5.10^7^ cells/ml) (Fig 1B). Contrariwise, filamentation in the presence of CPT was very strong when the wild type strain was inoculated at low density in unconditioned medium supplemented with the same glucose concentration (~ 3%) remaining in the conditioned medium (Fig 1B). Accordingly, the average cell length of CPT-treated wild type cells incubated for 6h in unconditioned medium became progressively reduced when incubated in conditioned medium obtained from 5.10^6^, 5.10^7^, and 10^8^ cells/ml density cultures (43.8 ± 14,6 μm *versus* 21.6 ± 11.6, 14.4± 5.5 and 11.4± 1.9 μm, respectively; Fig 1C). *S*. *japonicus* strains produce a readily visible mycelium when cultured for several days in a malt extract-based solid medium (YEMA) [19]. This provides an alternative biological readout to CPT treatment to explore a putative negative role of QS during hyphal growth progression in response to nutritional changes. As compared to unconditioned medium, the mycelial area expansion of wild type cells in YEMA plates also decreased progressively when supplemented with conditioned medium obtained from 5.10^6^ cells/ml or 5.10^7^ cells/ml density cultures (Fig 1D).

Several acyclic and/or aromatic alcohols, such as farnesol, tyrosol, phenylethanol and tryptophol have been described to mediate QS in yeasts species from the genera *Candida*, *Debaryomyces* and *Saccharomyces* [22, 23]. To identify the QS molecule/s responsible for the inhibition of *S*. *japonicus* hyphal growth at high population densities, the conditioned medium obtained from a stationary phase culture was extracted with organic solvents, and the lipophilic compounds were separated, identified, and quantified by GC/MS, HPLC/MS and/or HPLC/UV analysis. This experimental approach, which included a comparative analysis of chemically synthesized standards, revealed the presence of different peaks that were unequivocally identified as phenylethanol, tyrosol, and tryptophol (S1 Fig and Methods). The accumulation of these aromatic alcohols in the culture medium was growth phase-dependent, and reached a maximum during stationary phase (~100 μM for phenylethanol, ~75 μM for tyrosol, and ~10 μM for tryptophol; Fig 1E). We found that the cell length average of CPT-treated wild type cells after 6h of incubation in rich medium at low population density (initial inoculum of 10^6^ cells/ml) became significantly reduced in the presence of 1 mM phenylethanol and/or tryptophol, but not with tyrosol (Fig 1F). Furthermore, the growth of *S*. *japonicus* wild type cells was not altered in the presence of ≤ 2 mM of the above aromatic alcohols in the growth medium (S2 Fig). As compared to untreated medium, the mycelial area expansion of wild type cells in YEMA plates was significantly decreased in the presence of 1 mM of either phenylethanol or tryptophol, and further reduced with a combination of both alcohols (Fig 1G). Contrariwise, mycelium expansion was not negatively affected by the addition of tyrosol (Fig 1G). Virtually identical results were obtained after incubation in YES solid media plates supplemented with 10% red grape extract (RGE medium), which has been recently shown to induce a strong mycelial development in *S*. *japonicus* (Fig 1G) [27]. Altogether, these results demonstrate the existence of a QS mechanism mediated by the aromatic alcohols phenylethanol and tryptophol in *S*. *japonicus* that represses dimorphic yeast to hypha differentiation in response to increased population density.

### The stress-responsive functions of the SAPK pathway are conserved in *S*. *japonicus*

Sty1, the key member of the SAPK pathway, controls multiple cellular events in response to environmental cues in *S*. *pombe* [2]. An amino-acid sequence comparison (ClustalW) revealed that the putative MAPK Sty1 in *S*. *japonicus* (Gene ID: SJAG_02592) shows ~94% identity with *S*. *pombe* Sty1 (S3 Fig). This includes residues involved in ATP binding, the proton acceptor site, MAPKK (-DXXD- motif) and common (-ED- motif) docking sites, as well as the -TGY- activation loop present in MAP kinases of the p38 type (S3 Fig) [28]. By employing anti-Hog1 and phospho-p38 antibodies that specifically detect the respective total and dually phosphorylated isoforms in yeast MAPKs of the p38 type [29], a single band of the predicted molecular weight (~42 kDa) was detected with both antibodies in extracts from exponentially growing *S*. *japonicus* wild type cells (S3 Fig). These signals were totally absent in extracts from a *S*. *japonicus* strain lacking the putative Sty1 ORF via homologous recombination (*sty1Δ*; S3 Fig), thereby confirming that it encodes the sole p38-type MAPK present in this organism.

**Fig 2:**
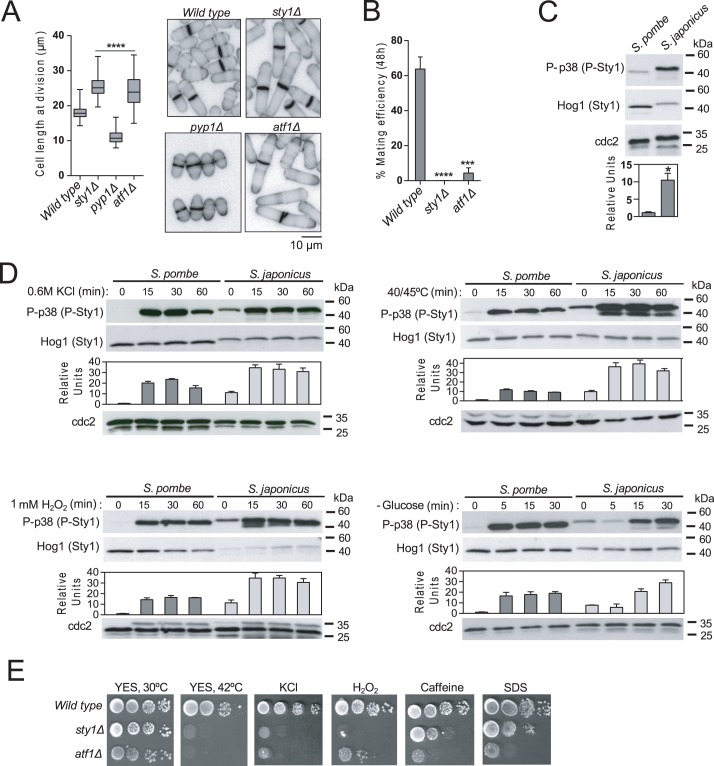
The stress-regulatory functions of the SAPK pathway are conserved in *S*. *japonicus*. (A) Cell length at division represented as box and whisker plots in *S*. *japonicus* wild type, *sty1Δ*, *pyp1*Δ and *atf1*Δ mutants. Experiment was performed per triplicate (n≥ 200 cells/strain) and quantification of one is shown. ****, *P*<0.0001, as calculated by unpaired Student´s *t* test. Cell morphology of each strain was analyzed by fluorescence microscopy after staining with calcofluor white. Scale bar: 10 μm. (B) *S*. *japonicus* wild type, *sty1Δ* and *atf1Δ* mutants of the h^+^ mating type were mixed with wild type h^-^ cells, poured on EMM2-N plates, and incubated at 28°C. The percentage of conjugation efficiency (as mean ± SD) was determined after 24h of incubation by microscopic counting of number of vegetative cells, zygotes, and asci. Biological triplicate samples (n≥300 cells) were counted for each cross. ***, *P*<0.005; ****, *P*<0.001, as calculated by unpaired Student´s *t* test. (C) Wild type *S*. *pombe* and *S*. *japonicus* strains were grown in YES medium to mid-log phase. Activated/total Sty1 were detected with anti-phosho-p38 and anti-Hog1 antibodies, respectively. Anti-cdc2 was used as loading control. Relative units as mean ± SD (biological triplicates) for Sty1 phosphorylation (anti-phosho-p38 blot) were determined with respect to the internal control (anti-Hog1 blot). *, *P*<0.05, as calculated by unpaired Student´s *t* test. (D) Wild type *S*. *pombe* and *S*. *japonicus* strains were grown in YES medium to mid-log phase, and treated with either 0.6 M KCl (upper left panel), 1 mM H_2_O_2_ (lower left panel), incubated at 40°C (*S*. *pombe*) or 45°C (*S*. *japonicus*) (upper right panel), and shifted to the same medium without glucose and supplemented with 3% glycerol (lower right panel). Activated/total Sty1 were detected with anti-phosho-p38 and anti-Hog1 antibodies, respectively. Anti-cdc2 was used as loading control. Relative units as mean ± SD (biological triplicates) for Sty1 phosphorylation (anti-phosho-p38 blot) were determined with respect to the internal control (anti-Hog1 blot). (E) Serially diluted cells of wild type, *sty1*Δ and *atf1*Δ strains were spotted on YES plates supplemented with either 1.2 M KCl, 1.5 mM H_2_O_2_, 6 mM caffeine, or 0.01% SDS, and incubated for 3 days at either 30 or 42°C. Results representative of three independent experiments are shown.

In *S*. *pombe* Sty1 positively regulates cell cycle progression during the G2/M transition, the initiation of sexual differentiation and the chronological lifespan [30–32]. *S*. *japonicus* s*ty1Δ* cells also display elongated cell length at division as compared to control cells (25.5 ± 2.5 *vs* 18.8 ± 1.8 μm; Fig 2A), and either h^+^ or h^-^ heterotallic kinase-deleted mutants were totally defective for mating when crossed with wild type cells of the opposite mating types (Fig 2B). In addition, *S*. *japonicus* s*ty1Δ* cells quickly lost viability in stationary phase cultures after 2–3 days of growth in rich medium as measured by either phloxine B staining or growth plate assays (S4 Fig). The relative total levels of Sty1 in unperturbed exponentially growing *S*. *japonicus* cells were approximately half of those present in *S*. *pombe*. However, basal Sty1 phosphorylation in *S*. *japonicus* was significantly increased as compared to that of *S*. *pombe* (Fig 2C), and maintained relatively constant during the growth curve at cellular densities ranging from 2.10^6^ to 2.10^8^ cells/ml (S3 Fig). The specific tyrosine phosphatase Pyp1 is the main negative regulator of basal Sty1 phosphorylation in *S*. *pombe* [2]. Similar to the equivalent *S*. *pombe* mutant, *S*. *japonicus* cells with a deletion in the Pyp1 ortholog (gene ID SJAG_02013) displayed a clear reduction in cell length at division as compared to the wild type strain (11.0 + 0.2 μm; Fig 2A). Hence, the role of Pyp1 as a negative regulator of Sty1 function may be conserved in both fission yeast species. *S*. *pombe* Sty1 is activated in response to multiple stress conditions such as heat shock, saline, or oxidative stress, as well as glucose deprivation, in order to promote cellular adaptation to environmental changes (Fig 2D) [2, 33]. Similarly, *S*. *japonicus* Sty1 became highly activated when cultures were incubated at 45°C (heat shock), treated with 0.6 M KCl (salt stress), after addition of 0.5 mM hydrogen peroxide (oxidative stress), or starved from glucose (Fig 2D). The growth sensitivity of *S*. *pombe sty1Δ* cells in response to high temperature, saline stress (KCl), oxidative stress (hydrogen peroxide), caffeine and SDS [2, 33, 34], was also shared by the *S*. *japonicus sty1Δ* mutant (Fig 2E). The bZIP domain protein Atf1 is the main transcription factor regulated downstream by Sty1 in *S*. *pombe* [35]. A Blast search revealed the presence of a single Atf1 ortholog in *S*. *japonicus* genome (Gene ID: SJAG_00266). *S*. *japonicus* Atf1 shows an overall ~43.5% amino acid identity with *S*. *pombe* Atf1 (S3 Fig). The HRA, osmotic stress, and basic-leucine zipper domains involved in recombination, stress response, and DNA binding, respectively [36], are strongly conserved in *S*. *japonicus* Atf1 with regard to the *S*. *pombe* counterpart (88, 61.3, and 95.3% identity, respectively), and also includes several of the putative MAPK-dependent phoshorylation sites (SP/TP) present in *S*. *pombe* Atf1 (S3 Fig). Remarkably, a *S*. *japonicus* mutant lacking the Atf1 ortholog (*atf1Δ* strain) showed a defective G2/M progression similar to that of the *sty1Δ* mutant (cell length at division: 24.3 ± 4.5 μm; Fig 2A). As compared to *sty1Δ* cells, *S*. *japonicus atf1Δ* cells were slightly less defective during mating (Fig 2B), and showed a significant growth sensitivity in response to stress (Fig 2E). The above results indicate that the SAPK pathway plays a critical role in the regulation of cell cycle progression, the sexual differentiation, the chronological lifespan, and the general cellular adaptive response to environmental stress in *S*. *japonicus*. They also suggest that many of these functions may rely on the transcriptional activity mediated by Atf1.

### SAPK function negatively modulates induction and progression of hyphal growth in *S*. *japonicus*

**Fig 3:**
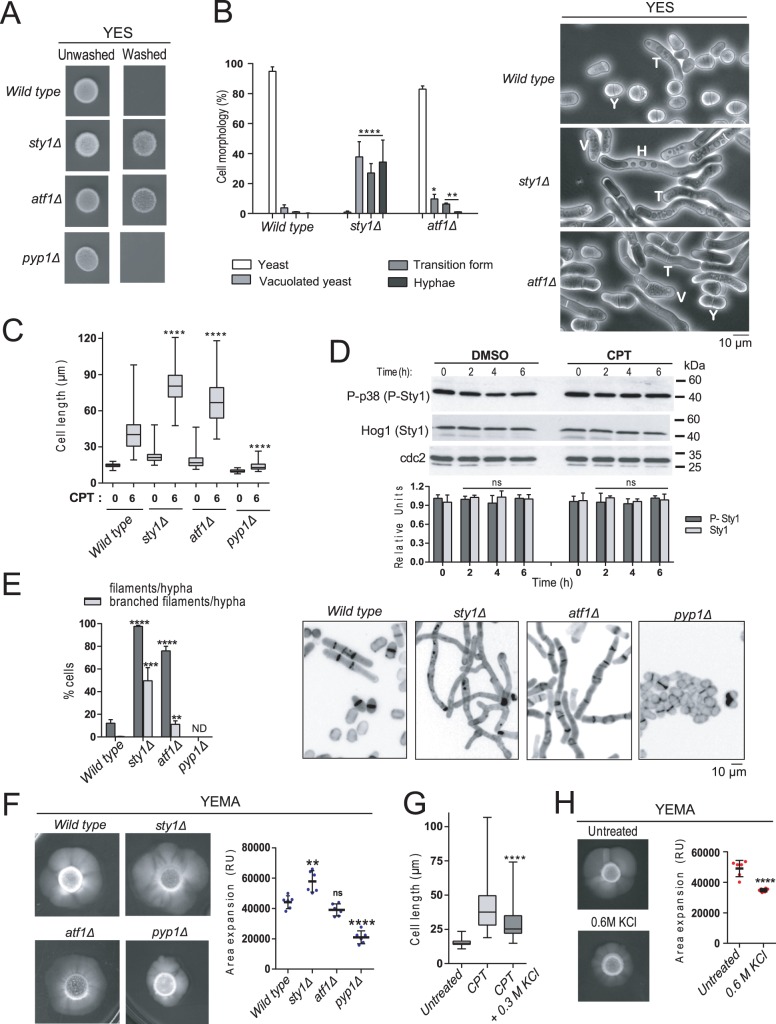
The SAPK pathway negatively regulates hyphal growth in *S*. *japonicus*. (A) Cells (~10^6^) of wild type, *sty1*Δ, *atf1*Δ and *pyp1*Δ strains were spotted on YES plates, incubated for 2 days at 30°C, and photographed before and after washing extensively (30 sec) with distilled water. Results representative of three independent experiments are shown. (B) Cells of the above strains were grown in YES plates for 12 h at 30°C, recovered by extensive washing with YES medium, and observed by phase-contrast microscopy. Quantification (expressed as percentage) of yeasts (Y), vacuolated yeasts (V), transition forms (T) and hyphae (H) present in each culture is shown in the left panel. Percentages are expressed as mean ± SD and correspond to biological triplicates (n≥200 cells/sample). *, *P*<0.05; **, *P*<0.005; ****, *P*<0.0001, as calculated by unpaired Student´s *t* test. Representative phase-contrast micrographs different cell morphologies found for each strain are shown in the right panel. Scale bar: 10 μm. (C) Cell length represented as box and whisker plots in *S*. *japonicus* wild type, *sty1*Δ *atf1*Δ, and *pyp1*Δ mutants growing exponentially in high glucose (6%) YES medium for 6h in the absence or presence of 0.2 μM CPT. Experiment was performed per triplicate (n≥ 200 cells) and quantification of one is shown. ****, *P*<0.0001, as calculated by unpaired Student´s *t* test. (D) Wild type *S*. *japonicus* strain growing exponentially (10^6^ cels/ml) in high glucose (6%) YES medium remained untreated (DMSO; negative control) or treated with 0.2 μM CPT for the indicated times. Activated/total Sty1 were detected with anti-phosho-p38 and anti-Hog1 antibodies, respectively. Anti-cdc2 was used as loading control. Relative units as mean ± SD (biological triplicates) for Sty1 phosphorylation (dark grey bars) and total Sty1 levels (light grey bars) were determined with respect to the internal control (anti-cdc2 blot). (E) Wild type, *sty1*Δ, *atf1*Δ, and *pyp1*Δ strains were grown in high glucose (6%) YES medium with 0.2 μM CPT for 24h, and the percentage of filaments/hyphae (dark grey bars) and branched filaments/hyphae (light grey bars) were quantified. Percentages are expressed as mean ± SD and correspond to biological triplicates (n≥200 cells/sample). **, *P*<0.005; ***, *P*<0.001; ****, *P*<0.0001, as calculated by unpaired Student´s *t* test. The right panels show the representative cell morphology of each strain observed by fluorescence microscopy after staining with calcofluor white. Scale bar: 10 μm. (F) Cells from log-phase cultures of the indicated strains growing in YES medium (2.10^6^) were spotted on YEMA plates, incubated at 30°C for 7 days, and then photographed. The total area of mycelial expansion (expressed as relative units) was measured for each strain (n≥6) and represented as scatter plot. **, *P*<0.005; ****, *P*<0.001; ns, not significant, as calculated by unpaired Student´s *t* test. (G) Exponentially growing *S*. *japonicus* wild type cells were inoculated at an initial cell density of 10^6^ cells/ml in high glucose (6%) YES medium and incubated for 6h with 0.2 μM CPT without further treatment or supplemented with 0.3 M KCl. Cell length is represented as box and whisker plots. Data obtained after quantification of one experiment performed per triplicate (n≥ 200 cells/sample) is shown. ****, *P*<0.0001, as calculated by unpaired Student´s *t* test. (H) Cells from a log-phase culture of wild type strain growing in YES medium (2.10^6^) were spotted on YEMA and YEMA+0.6 M KCl plates, incubated at 30°C for 7 days, and then photographed. The total area of mycelial expansion was measured (n≥6) and represented as scatter plot. ****, *P*<0.001, as calculated by unpaired Student´s *t* test.

When growing in solid rich medium (YES-agar; 24h) *S*. *japonicus sty1Δ* and *atf11Δ* mutants displayed a wash-resistant invasive phenotype that is absent in wild type and *pyp1Δ* cells (Fig 3A), suggesting the existence of an altered developmental and/or growth pattern. *S*. *japonicus* morphological transition from yeast to hypha during inducing conditions (i.e. after CPT treatment or in RGE medium) involves three successive stages that are known as vacuolated yeast (bipolar growing cells with numerous cytoplasmic vacuoles), transition forms (monopolar growing cells with numerous small vacuoles at the non-growing end), and hyphae (large monopolar growing cells with 1–2 large vacuoles at the non-growing end) [16, 27]. Remarkably, as compared to wild type cells, both SAPK mutants, and in particular the *sty1Δ* mutant, showed a significant increase in the number of vacuolated yeast, transition forms and/or hyphae after 12 hours of growth under non-inducing conditions rich YES-agar plates (Fig 3B). The average cell length of filamentous *sty1Δ* and *atf1Δ* cells after 6h of incubation in rich medium supplemented with 0.2 μM CPT was significantly higher than in wild type cells (81,5 ± 15,4 μm and 67,3 ± 16,6 μm in *sty1Δ* and *atf1Δ* cells *versus* 42,6 ± 16 μm in wild type cells; Fig 3C), whereas differentiation in *pyp1Δ* cells became strongly impaired (13,9 ± 3,6 μm; Fig 3C). Wild type, *sty1Δ*, *atf1Δ*, and *pyp1Δ* mutants grew normally at those CPT concentrations (S4 Fig). Moreover, total and activated Sty1 levels remained unchanged and irrespective of the presence or absence of CPT in the medium (Fig 3D). Thus, in *S*. *japonicus* the SAPK pathway appears to negatively impact the induction of hyphal differentiation in a constitutive fashion. Prolonged incubation in the presence of CPT (24h) produced a much higher percentage of filaments and/or hypha in *sty1Δ* and *atf1Δ* mutants than in wild type cells (~98% and ~75% versus ~12%, respectively), while they remained absent in *pyp1Δ* cells (Fig 3E). Interestingly, *sty1Δ* hyphae were highly branched as compared to those from the *atf1Δ* mutant (~50% *versus* ~10%, respectively) (Fig 3E), suggesting that Sty1 function, but not Atf1, negatively affects the later stages of hyphal differentiation. Congruent with the above prediction, the *sty1Δ* mutant showed a significant increase in the mycelial area of expansion with respect to either wild type or *atf1Δ* cells when incubated in YEMA plates (Fig 3F). Contrariwise, mycelium production under the above conditions was strongly reduced in *pyp1Δ* cells (Fig 3F). Hence, in *S*. *japonicus* the SAPK pathway effectors Sty1 MAPK and Atf1 transcription factor downregulate the initiation of yeast to hypha transition in response to environmental cues, and Sty1 additionally represses the later stages of hyphal differentiation in an Atf1-independent fashion. Moreover, our results indicate that Sty1 phosphorylation must be maintained under a certain threshold to allow efficient yeast to hypha differentiation, and suggest that an increase in MAPK activity may result in a complete inhibition of this process. Indeed, treatment of low density *S*. *japonicus* cultures with either 0.3 M or 0.6 M KCl that hyperactivate Sty1 (Fig 2D), reduced hyphal differentiation in the presence of CPT (Fig 3G), and mycelial expansion in YEMA plates, respectively (Fig 3H).

### *S*. *japonicus* QS is attenuated in the absence of SAPK function

**Fig 4:**
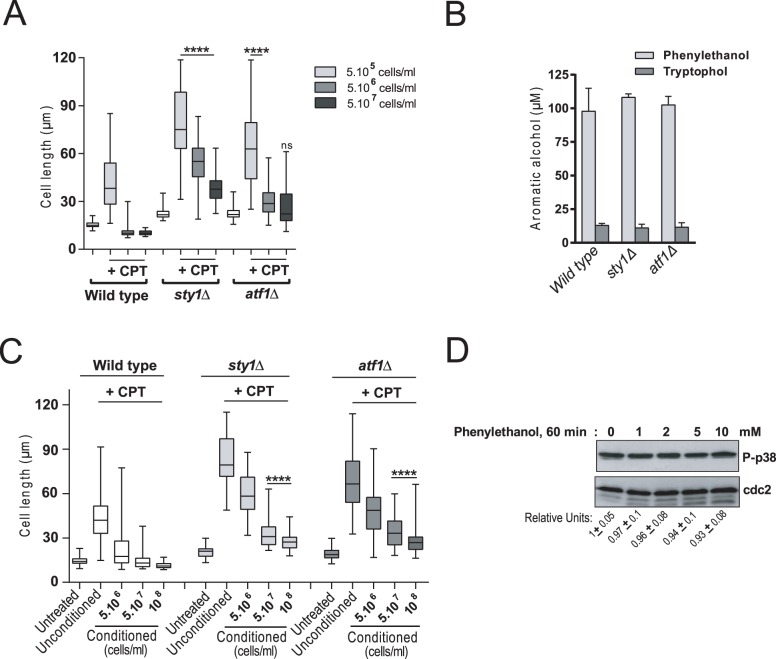
*S*. *japonicus* QS is partially suppressed in the absence of SAPK function. (A) Exponentially growing *S*. *japonicus* wild type, *sty1*Δ, and *atf1*Δ cells were inoculated at the initial cell densities of 5.10^5^, 5.10^6^, or 5.10^7^ cells/ml in high glucose (6%) YES medium, and incubated for 6h in the absence or presence of 0.2 μM CPT. Cell length is represented as box and whisker plots. Data obtained after quantification of one experiment performed per triplicate (n≥ 200 cells/strain) is shown. ****, *P*<0.0001; ns, not significant, as calculated by unpaired Student´s *t* test. (B) Media supernatants were recovered by filter-sterilization of stationary phase cultures of wild type, *sty1*Δ, and *atf1*Δ strains, and the concentration of phenylethanol (light grey bars) and tryptophol (dark grey bars) secreted into the medium was determined by GC/MS or HPLC/MS analysis. Data are expressed as mean ± SD and correspond to biological triplicates. (C) Cell length represented as box and whisker plots in *S*. *japonicus* wild type, *sty1*Δ, and *atf1*Δ strains cells growing exponentially for 6h in the presence of 0.2 μM CPT in either unconditioned or conditioned YES medium (3% glucose) obtained from a culture of the wild type strain growing to a density of 5.10^6^, 5.10^7^, or 10^8^ cells/ml. The experiment was performed per triplicate (n≥ 200 cells) and quantification of one is shown. ****, *P*<0.0001, as calculated by unpaired Student´s *t* test. (D) Wild type *S*. *japonicus* strain growing exponentially (10^6^ cells/ml) in high glucose (6%) YES medium remained untreated (DMSO; negative control) or treated with 1, 2, 5 or 10 mM phenylethanol for 60 minutes. Activated Sty1 was detected with anti-phosho-p38, while anti-cdc2 was used as loading control. Relative Sty1 activation units as mean ± SD (biological triplicates) were determined with respect to the internal control (anti-cdc2 blot).

The observation that a QS mechanism and the SAPK pathway negatively regulate the induction and progression of *S*. *japonicus* hyphal growth prompted us to analyze the possible functional relationship between both pathways. We found that *sty1Δ* and, to a lesser extent, *atf1Δ* cells were able to differentiate into filaments to some extent after 6h of incubation in the presence of 0.2 μM CPT when inoculated at an initial cell density of 5.10^6^ cells/ml that completely blocks hyphal differentiation in wild type cells (Fig 4A). This behavior was observed in the *sty1Δ* mutant even at higher densities of 5.10^7^ cells/ml (Fig 4A). The ability of *sty1Δ* and *atf1Δ* mutants to partially suppress cell density dependent inhibition of hyphal differentiation was not due to a defective production of QSMs, since the levels of phenylethanol and tryptophol in the respective cultures were similar to those of wild type cells (Fig 4B). Moreover, and in agreement with the above results, CPT-treated *sty1Δ* and *atf1Δ* mutants were able to partially differentiate into elongated forms when incubated in conditioned medium obtained from 5.10^7^ and 10^8^ cells/ml density cultures, which strongly inhibits the morphological transition in wild type cells (Fig 4C). Finally, exogenous addition of increased concentrations of QSMs (phenylethanol from 1 to 10 mM; 60 min) did not change basal Sty1 phosphorylation status in exponentially growing *S*. *japonicus* cultures (Fig 4D). Altogether, these observations further confirm the biological relevance in *S*. *japonicus* of the SAPK pathway as a constitutive repressor of hyphal development that allows QS to operate over a specific cell density threshold.

### Nrg1 is an activator of hyphal growth in *S*. *japonicus* whose basal and induced expression is repressed by the SAPK pathway and QS

**Fig 5:**
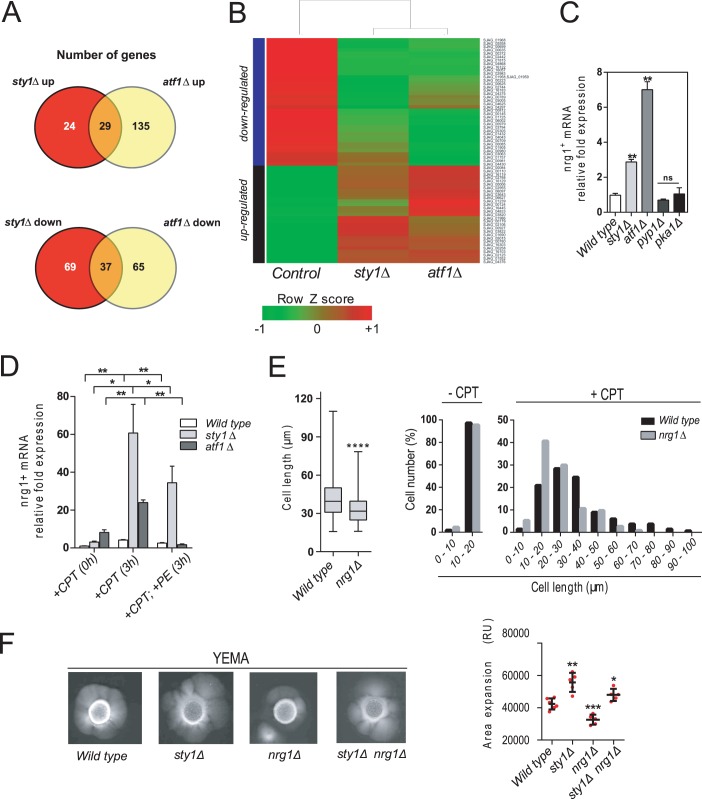
Nrg1 activates hyphal growth in *S*. *japonicus* and is repressed by QS and the SAPK pathway. (A) Venn diagrams indicating the number of differentially expressed up- and downregulated genes during unperturbed growth in *sty1*Δ and *atf1*Δ mutants with respect to wild type cells. (B) Heatmap of the subset of differentially expressed genes (up- and downregulated) during unperturbed growth in *sty1*Δ and *atf1*Δ mutants with respect to wild type cells. Green indicates decrease and red indicates increase in gene expression. (C) *nrg1*^*+*^ mRNA levels were measured by qPCR from total RNA extracted from cell samples corresponding to *S*. *japonicus* wild type, *sty1*Δ, *atf1*Δ, *pyp1*Δ, and *pka1*Δ strains growing exponentially in YES medium. Results are shown as relative fold expression (mean ± SD) from three biological repeats. **, *P*<0.005; ns, not significant, as calculated by unpaired Student´s *t* test. (D) *nrg1*^*+*^ mRNA levels measured by qPCR from total RNA extracted from cell samples from wild type, *sty1*Δ and *atf1*Δ strains growing exponentially (time 0h), incubated for 3h in the presence of 0.2 μM CPT, or for 3h in the presence of 0.2 μM CPT plus 0.5 mM phenylethanol (PE). Results are shown as relative fold expression (mean ± SD) from three biological repeats. *, *P*<0.05; **, *P*<0.005; as calculated by unpaired Student´s *t* test. (E) Cell length represented as box and whisker plots in *S*. *japonicus* wild type, and *nrg1*Δ mutants growing exponentially in high glucose (6%) YES medium for 6h in the presence of 0.2 μM CPT. Experiment was performed per triplicate (n≥ 300 cells) and quantification of one is shown. ****, *P*<0.0001, as calculated by unpaired Student´s *t* test. (F) Cells from log-phase cultures of the indicated strains growing in YES medium (2.10^6^) were spotted on YEMA plates, incubated at 30°C for 7 days, and then photographed. The total area of mycelial expansion (expressed as relative units) was measured for each strain (n≥6) and is represented as scatter plot. *, *P*<0.05; **, *P*<0.005; ***, *P*<0.001; as calculated by unpaired Student´s *t* test.

Our findings suggest that the SAPK pathway through the Sty1–Atf1 branch may act transcriptionally to repress hyphal initiation in *S*. *japonicus*. To obtain further insight into this hypothesis, we performed a transcriptome analysis via high-coverage RNA sequencing (RNAseq). We thereby specifically searched for differentially expressed genes in *sty1Δ* and *atf1Δ* mutants up- and down-regulated as compared to wild type cells, and whose products might positively or negatively regulate yeast to hypha dimorphic switch. Two biological replicates were tested for global gene expression in wild type *versus sty1Δ* and *atf1Δ* strains growing in rich medium at the early exponential growth phase. As shown in Fig 5A, transcript levels of 29 genes from a total of 188 (~15%) were increased more than twofold (log_2_ fold change ≥ 1) in both *sty1Δ* and *atf1Δ* mutants as compared to wild type cells, whereas 37 genes from a total of 171 (~22%) were downregulated (log_2_ fold change ≤ 1) in both mutants. Contrariwise, only 5 (~3%) and 14 (~5%) genes were up- and downregulated, or down- and upregulated, respectively, in both *sty1Δ* and *atf1Δ* mutants. Hence, while there is a relatively low level of similarity in gene expression changes between *sty1Δ* and *atf1Δ* cells during unperturbed growth, shared induction or repression of common genes is enriched in both mutants. A heatmap of the subset of common up- and downregulated genes revealed evident differences in expression between *sty1Δ* and *atf1Δ* mutants (Fig 5B). The complete gene list of specific and common up- and downregulated genes is shown in S1–S8 Tables. Validation of RNAseq data was made through qPCR, for which a set of six significantly differentially expressed genes up- and downregulated in *sty1Δ* and *atf1Δ* mutants as compared to wild type cells was confirmed (S5 Fig). Approximately half of the identified common up-regulated genes encode putative hypothetical proteins without assigned function (S1–S8 Tables). The remaining 15 genes were functionally categorized by gene ontology (GO) terms and include, among others, urea, glucose, and amino acids plasma membrane transporters, as well as others involved in the oxidation-reduction process (S6 Fig). Similarly, half of the common down-regulated genes encode either fungal and/or hypothetical proteins without assigned function whereas the other 23 are functionally diverse, including genes involved in oxidation-reduction mechanisms, cell wall ascospore formation, DNA metabolism, and transcription (S6 Fig).

Among the genes whose mRNA expression is induced in both *sty1Δ* and *atf1Δ* mutants we identified *nrg1*^*+*^, which encodes a putative ortholog of the C_2_H_2_ zinc finger transcriptional repressor Nrg1 that negatively regulates, respectively, pseudohyphal growth and yeast to hypha dimorphism in *S*. *cerevisiae* and *C*. *albicans* [25, 26, 37]. *S*. *japonicus* Nrg1 is a putative 217 amino-acids protein with a low level of overall sequence identity with *C*. *albicans* Nrg1 (~27%), that rises to ~57% within the 50 amino-acids C_2_H_2_ zinc finger region (S7 Fig). qPCR analysis confirmed that *nrg1+* mRNA levels increase ~3 to ~7 fold in exponentially growing *sty1Δ* and *atf1Δ* mutants, respectively, as compared to wild type cells (Fig 5C). Contrariwise, *nrg1+* mRNA levels were modestly reduced in the *pyp1Δ* mutant (Fig 5C). The cAMP-PKA pathway-activated transcriptional down-regulation of Nrg1 expression promotes yeast to hypha transition in *C*.*albicans* [38]. However, the *nrg1*^*+*^ mRNA levels in a *S*. *japonicus* mutant lacking the single Pka1 catalytic subunit (*pka1Δ*) [39] were similar to those present in wild type cells when growing in glucose-rich medium (Fig 5C), suggesting that the cAMP-PKA pathway does not regulate Nrg1 expression in this organism. As compared to untreated cells, *nrg1*^*+*^ expression increased in wild type cells after 3h in the presence of CPT, and this rise was much more evident in both *sty1Δ* and *atf1Δ* mutants (Fig 5D). Moreover, exogenous addition of QSMs (phenylethanol 0.5 mM) partially reduced the enhanced expression of *nrg1*^*+*^ in CPT-treated control and *sty1Δ* cells, and quite strongly in *atf1Δ* cells (Fig 5D). Therefore, Nrg1 expression may be repressed via two different/independent mechanisms, one involving Sty1-Atf1, and another mediated by QS.

The above findings draw a scenario where *S*. *japonicus* Nrg1 is an activator rather than a repressor of hyphal growth. In support of this hypothesis, we found that both the average cell length and size distribution of filamentous *nrg1Δ* cells after 6h of incubation with 0.2 μM CPT was lower than in wild type cells (34 ± 12,1 μm in *nrg1Δ* cells *versus* 43,8 ± 18,4 μm in wild type cells) (Fig 5E). Importantly, this phenotype was accompanied by a significant reduction in the mycelial area expansion in *nrg1Δ* cells as compared to wild type cells (Fig 5F). Indeed, simultaneous deletion of Nrg1 in *sty1Δ* cells (*nrg1Δ sty1Δ* double mutant) reduced, but did not suppress, the increased mycelial expansion shown by the *sty1Δ* single mutant (Fig 5F). Hence, the SAPK pathway may limit Nrg1 function as an activator of hyphal differentiation in *S*. *japonicus*.

## Discussion

QS mediates developmental responses in several fungal species within the phylum Ascomycota, including the subphyla Pezizomycotina (several *Aspergillus* species) and Saccharomycotina (budding yeasts *S*. *cerevisiae* and *Debaryomyces hansenii*) [22, 23]. The demonstration of a QS brought about by aromatic alcohols that inhibits *S*. *japonicus* hyphal development is, to our knowledge, the first description of such a mechanism within the fission yeast clade (subphylum Taphrinomycotina). The yeast to hypha transition in response to CPT is blocked in *S*. *japonicus* when the initial inoculum is ≥ 2.10^6^ cells/ml, a value fairly similar to that of *C*. *albicans*, which develops into filamentous forms during inducing conditions at densities ≤10^6^ cells/ml [22, 23]. Importantly, *S*. *japonicus* hyphal growth is strongly abolished in conditioned medium obtained from high cell density cultures. An exhaustive compositional analysis of this medium identified phenylethanol and tryptophol as the main QSMs of *S*. *japonicus*. Both compounds satisfy the main criteria proposed to classify a molecule/s as true QSMs [22, 23]. They accumulate during the growth curve in a density-dependent manner until reaching a maximum during the stationary phase. Moreover, their exogenous addition limited yeast to hypha transition in liquid medium and reduced the mycelial expansion in solid medium. Phenylethanol and tryptophol act as QSMs in *S*. *cerevisiae*, where they positively control pseudohyphal and invasive filamentous morphology during nitrogen starvation [40]. Although similarly produced in response to environmental changes, our findings suggest that the biological responses induced by the above QSMs have evolved differently in these fungal species. Phenylethanol and tryptophol are also produced by *C*. *albicans* in addition to farnesol, the major QSM that represses hyphal development in this organism, but their putative role as QSMs is not clear [22, 23]. Repeated attempts failed to detect farnesol in *S*. *japonicus* conditioned medium. However, we found out that addition of increased concentrations of this aromatic alcohol that do not interfere with cell growth (5 to 40 μM), significantly blocked *S*. *japonicus* hyphal differentiation induced with CPT, and were able to reduce hyphal expansion in YEMA solid plates (S8 Fig). Hence, farnesol behaves similarly as a QSM than the naturally produced phenylethanol and tryptophol to block hyphal formation in this organism. The amount of chemically synthesized/purified phenylethanol and tryptophol that elicit the QS mechanism (500–1000 μM) is higher than the maximal concentrations of both compounds secreted by *S*. *japonicus* into rich medium (~10 to 100 μM). This discrepancy, as reported in other fungal species like *C*. *albicans* or *Cryptococcus neoformans* [41], might be related to either different physicochemical and compositional nature of the conditioned medium (pH, nitrogen source,‥), or to the presence of other unknown metabolites that might synergize the QS effect of both aromatic alcohols. *S*. *japonicus* releases important amounts of tyrosol into the growth medium (~75 μM). However, similarly to *S*. *cerevisiae* [40], this molecule does not appear to play a noticeable role during hyphal development.

The SAPK MAPK cascade governs multiple cellular events during vegetative growth and in response to environmental changes in *S*. *pombe*. Many of these functions, such as the cellular adaptation to stress conditions and the sexual differentiation, are executed transcriptionally by Atf1, which becomes phosphorylated and stabilized by activated Sty1 [42, 43]. Other roles are mostly prompted in a transcription-independent manner and depend upon the ability of Sty1 to either activate or inhibit different substrates such as cell cycle kinases, mRNA binding proteins, and translation factors [2, 30]. Like the equivalent *S*. *pombe* mutants, *S*. *japonicus* s*ty1Δ* and *atf1Δ* mutants are virtually sterile, and quickly lose viability when reaching the stationary phase, indicating that the SAPK pathway positively regulates sexual differentiation and chronological lifespan. Although showing a higher basal activity than in *S*. *pombe* during unperturbed growth, Sty1 is strongly activated in *S*. *japonicus* in response to multiple stress situations, and s*ty1Δ* and *atf1Δ* mutants are growth sensitive under these conditions. Hence, even though *S*. *japonicus* displays some distinctive biological features with regard to *S*. *pombe* [6–10], the core stress-responsive functions of the SAPK pathway appear to be evolutionary conserved in both *Schizosaccharomyces* species. However, an unexpected exception to this rule is the finding that, contrary to the *S*. *pombe* ortholog [44], Atf1 may positively regulate G2/M progression in *S*. *japonicus*, since *atf1Δ* strain showed an increased cell size at division similar to that of the *sty1Δ* mutant.

In contrast to *S*. *pombe*, *S*. *japonicus* undergoes robust hyphal development in response to environmental cues [19]. Our results suggest that the SAPK pathway acts as a general negative regulator of yeast to hypha transition in this organism. This conclusion is supported by several findings, such as the increased presence of pseudohyphal cells and an invasive phenotype in *sty1Δ* and *atf1Δ* mutants, the increased length of the hyphae and branching in these mutants during hyphal initiation as compared to wild type cells, and the enhanced hyphal expansion displayed by the *sty1Δ* mutant in solid medium. Recently, it has been show that *S*. *japonicus* mutants lacking the Sty1 activator MAPKK Wis1, but not Sty1 itself, shows increased mycelial expansion [45]. This is a puzzling observation, since both kinases act in a linear pathway, and their deletion should therefore give rise to highly similar, if not identical, cellular phenotypes. Moreover, we found that cells lacking the tyrosine phosphatase Pyp1 that inactivates Sty1 were highly impaired in hyphal development under both conditions, thus confirming the general role of the SAPK pathway in the negative regulation of yeast to hypha transition.

In *S*. *japonicus* Sty1 and/or Atf1 appear to negatively regulate yeast to hyphal development both at the transcriptional and post-translational levels. In *S*. *pombe* Atf1 becomes phosphorylated by Sty1 during stress to induce the expression of CESR genes that modulate the adaptive cell response to the triggering stimuli [46]. However, a number of genes are also up- or down-regulated in *atf1Δ* cells under unperturbed conditions (low Sty1 activity) [46], which suggests that Sty1 activation threshold prompts Atf1 to function either as a repressor or as a transcriptional activator. This mechanism is likely conserved in *S*. *japonicus*, considering the similar activation pattern of Sty1 in response to stress, and the conserved phenotypes of *sty1Δ* and *atf1Δ* mutants. The fact that Sty1 activity is maintained at a basal level during the early stages of hyphal initiation, together with the observation that both *sty1Δ* and *atf1Δ* cells are derepressed for filamentation in absence of stimulus, indicate that the Sty1-Atf1 branch negatively controls the initial step of this developmental process transcriptionally and in a constitutive fashion. However, Atf1 function seems less relevant during hyphal growth and maintenance, since, contrary to the *sty1Δ* mutant, mycelial expansion is not enhanced in *atf1Δ* cells. Sty1 might negatively regulate the later stages of hyphal growth transcriptionally and independently of Atf1, at a post-translational level, or in both ways. The functional relationship between Sty1 and Atf1 during control of this transition in *S*. *japonicus* somehow resembles that between the Sty1 ortholog Hog1 and the transcriptional repressor Sko1 in *C*. *albicans*. In this organism the basal level of phosphorylated Hog1 represses the yeast to hypha development through Sko1 [47], which also serves as activator of genes induced by stress [47, 48]. Indeed, both Hog1 and Sko1 repress yeast-to hypha transition and *hog1* and *sko1* mutants display a hyperfilamentous phenotype under non-inducing conditions [49, 50]. In our model, the functional relevance of the SAPK pathway as a constitutive repressor of dimorphism was further confirmed as Atf1 or Sty1 deletion partially attenuated the QS mechanism that blocks hyphal development at high cell densities.

The transcriptional repressor Nrg1 is a key negative regulator of hyphal initiation and maintenance in *C*. *albicans*. whereas *nrg1* mutants constitutively grow as long pseudohyphae as the expression of hypha-specific genes is constitutively derepressed [25, 26]. Hyphal initiation in *C*. *albicans* requires a quick and temporary disappearance of Nrg1, whereas hyphal maintenance leads to exclusion of Nrg1 binding to promoters of hypha-specific genes through a mechanism involving reduction in Hog1 activity [38]. Remarkably, in *S*. *japonicus* the Nrg1 ortholog is not a repressor but an activator of hyphal differentiation, while the SAPK pathway may act as a major negative regulator of its expression and/or function. With respect to control cells *nrg1*^*+*^ expression is up-regulated in both *sty1Δ* and *atf1Δ* mutants during vegetative growth and hyphal initiation in response to CPT. Most important, Nrg1 absence elicited a reduction in cell filamentation in response to CPT, and also in the mycelial area of expansion of both wild type and *sty1Δ* cells. Therefore, repression of *nrg1*^*+*^ expression is biologically relevant during hyphal initiation and maintenance. However, despite the large increase in *nrg1*^*+*^ expression in the *atf1Δ* mutant with respect to the wild type cells, mycelial expansion is similar in both backgrounds, suggesting that Sty1 may also negatively regulate Nrg1 function post-translationally. Nrg1 amino-acid sequence contains several putative–SP/TP and -PXSP/TP- MAPK consensus phosphorylation sites (S7 Fig). It will be interesting to explore if whether Sty1 phosphorylates Nrg1 *in vivo* and the biological impact on its function. From a broader perspective, another important issue will be to elucidate the nature of the evolutionary structural and signaling constraints that define Nrg1to function either as activator (*S*. *japonicus*) or repressor (*C*. *albicans*) of hyphal growth in two distantly related yeast species. Importantly, QS represses Nrg1 expression independently of the SAPK pathway, as shown by the reduction in *nrg1*^*+*^ mRNA leves displayed by CPT-treated *sty1Δ* and *atf1Δ* cells in the presence of phenylethanol (QSM).

In conclusion, our results show that QS and SAPK signaling are major negative regulators of the dimorphic switch in *S*. *japonicus*. At low cellular densities the limited amount of QSMs in the growth medium make QS not functional, and the SAPK pathway negatively controls hyphal differentiation by downregulating elicitors of the yeast to hypha switch (Nrg1 and likely other factors) both transcriptionally and post-transcriptionally. This control is bypassed in response to specific environmental cues as yeast cells commit into hyphal growth. Contrariwise, increased presence of QSMs in high density cultures activates QS that blocks hyphal differentiation in response to those stimuli. *S*. *japonicus* positions as an alternative and suitable model organism to explore the intricate mechanisms regulating cellular differentiation in fungi.

## Methods

### Strains, growth conditions and reagents

The S. *japonicus* strains used in this work derive from the original isolates described by Niki *et al*. [18], and are listed in S9 Table. For comparative studies the wild type *S*. *pombe* strain 972 (h^-^) was employed. They were routinely grown with shaking at 30°C in rich (YES) or minimal (EMM2) medium with 2% glucose, and supplemented with adenine, leucine, histidine, or uracil (100 mg/L, Sigma Chemical)[51]. In osmotic-saline and oxidative stress experiments log-phase cultures (OD_600_ = 0.5; ~10^6^ cells/ml) were supplemented with either KCl (Sigma-Aldrich) or hydrogen peroxide (Sigma-Aldrich), respectively. In glucose starvation experiments cells grown in YES medium with 6% glucose were recovered by filtration, and resuspended in the same medium lacking glucose and osmotically equilibrated with 3% glycerol. Log-phase cultures grown in YES medium with 6% glucose were used in yeast to hyphae induction experiments with camptothecin (CPT, Sigma-Aldrich) supplemented to a final concentration of 0.2 μM. YEMA [16] and RGE [27] media with 2% agar were used to quantify mycelial growth. Chemically synthetized standards of phenylethanol, tyrosol, and tryptophol were obtained from Sigma-Aldrich. Conditioned media were prepared by inoculating cells from log-phase cultures (~10^6^ cels/ml) into fresh YES or YEMA medium to a final density of 5.10^6^, 5.10^7^, or 10^8^ cells/ml, incubated by 1–1.5h, and recovered by filtration.

### Gene disruption

Sequences of *S*. *japonicus* genes and those corresponding to *S*. *pombe* orthologs were obtained from the annotated database at *EnsemblFungi* (http://fungi.ensembl.org/Schizosaccharomyces_japonicus/Info/Index?db=core). The *S*. *japonicus sty1*^*+*^, *atf1*^*+*^, *pyp1*^*+*^, *pka1*^*+*^ and *nrg1*^*+*^, null mutants were obtained by entire deletion of the corresponding coding sequences by PCR-mediated strategy using plasmids pFK14 (*S*. *japonicus ura4*^*+*^ gene cloned into pGEMT-easy vector; [52]) or pFA6a-*natMX6* [53] as templates, and their replacement with either *ura4*^*+*^ or *natMX6* cassettes flanked by long 5´and 3´UTRs of respective genes following a PCR approach [54]. Oligonucleotides employed to obtain each one of the transformation cassettes are shown in S10 Table. *S*. *japonicus* transformation by electroporation was performed exactly as described [55].

### Quantification of mating efficiency

Equivalent amounts (~10^7^ cells) of strains of the opposing mating type were mixed, poured on EMM2 plates lacking nitrogen source (EMM2-N), and incubated at 28°C. The mating efficiency was determined after 24h of incubation by microscopic counting of number of vegetative cells (V), zygotes (Z), and asci (A), according to the following equation: % mating efficiency = (2Z+2A) x 100/(2Z+2A) + V. Triplicate samples (n≥300 cells) were counted for each cross.

### Plate assay of stress sensitivity for growth

*S*. *japonicus* wild type and mutant strains were grown in YES liquid medium to OD_600_ = 0.5, and appropriate decimal dilutions were spotted per duplicate on YES solid medium or in the same medium supplemented with varying concentrations of potassium chloride, hydrogen peroxide, caffeine, and sodium dodecyl sulphate (SDS; all purchased from Sigma). Plates were incubated at either 30 or 42°C for 3 days and then photographed. All the assays were repeated at least three times with similar results. Representative experiments are shown in the corresponding Figures.

### Quantification of mycelial growth during nutritional stress

Approximately 2.10^6^ cells from log-phase cultures (OD_600_ = 0.5) of wild type and mutant strains growing in YES medium were spotted on YEMA or RGE plates, incubated at 30°C for 7 days, and then photographed and saved as 16-bit .jpg digital images. The area of mycelial expansion was drawn by freehand for each strain (n≥6) and measured with the *analyze* tool using ImageJ [56].

### cDNA synthesis and quantitative real time polymerase chain reaction (qPCR)

*S*. *japonicus* wild type and mutant strains were grown in YES (6% glucose) in the absence or presence of CPT (0.2 μM) for the indicated times. Total RNAs were purified using the RNeasy mini kit (Qiagen), treated with DNase (Invitrogen), and quantitated using Nanodrop 100 spectrophotometer (ThermoScientific). Total RNAs (1 μg) were reverse transcripted into cDNA with the iScript reverse transcription supermix (BioRad). Quantitative real time polymerase chain reactions (qPCR) were performed using the iTaq Universal SYBR Green Supermix and a CFX96 Real‐Time PCR system (Bio‐Rad Laboratories, CA, USA). Relative gene expression was quantified based on 2^−ΔΔCT^ method and normalized using *leu1*^*+*^ mRNA expression in each sample. The list of gene-specific primers for qPCR is indicated in S10 Table.

### RNA sequencing and bioinformatics

High quality DNA-free total RNAs (two biological replicates each; RIN value >8.0) were purified from wild type, *sty1Δ* and *atf1Δ* mutants growing in YES medium to early log-phase (OD_600_ = 0.5). Total RNA was extracted using the Ambion PureLink RNA Mini Kit according to manufacturers instructions. Library construction and sequencing was performed by BaseClear, Netherlands. The Illumina TruSeq RNA-seq library preparation kit was used. Briefly, the mRNAs were purified by polyA capture, fragmented and converted to double-stranded cDNA. DNA adapters were ligated to both ends of the DNA fragments and subjected to PCR amplification. The sequence reads were generated using the Illumina HiSeq2500 system. FASTQ read sequence files were generated using bcl2fastq2 (version 2.18). Initial quality assessment was based on data passing the Illumina Chastity filtering. The second quality assessment was based on the remaining reads using the FASTQC quality control tool (version 0.11.5). Tophat2 (version 2.1.1) [57] has been used to align RNA-seq reads to the reference genome of *Schizosaccharomyces japonicus* (assembly: GCA_000149845.2 at ENA/EMBL). Cufflinks (version 2.2.1) [58] has been used to assemble the transcripts and estimate their abundances. The data analysis and the graphical representations have been done using an in-house R script. The NOISeq R package (version 2.24.0) [59] has been used for the differential expression tests. The results were filtered using q-value = 0,9 and FC = 2. The enrichment analysis was performed using the gProfileR R package (version 0.6.7) [60]. The *Schizosaccharomyces japonicus* GO annotation for biological processes has been retrieved from Ensembl Fungi version 41 [61] using the biomaRt R package (version 2.36.1) [62]. Complete RNAseq data are available from the European Nucleotide Archive (ENA) database (accession numbers ERS3040049, ERS3040050, ERS3040051, ERS3040052, ERS3040053, ERS3040054).

### Detection and quantification of total and activated Sty1 levels

In stress experiments cell extracts were prepared under native conditions employing chilled acid-washed glass beads and lysis buffer (10% glycerol, 50 mM Tris HCl pH 7.5, 15 mM Imidazole, 150 mM NaCl, 0.1% Nonidet NP-40, plus specific protease and phosphatase inhibitor, Sigma Chemical) [63]. In yeast to hypha experiments with CPT cell extracts were obtained by trichloroacetic acid precipitation as described in [64]. Dual phosphorylation in Sty1 was detected employing mouse monoclonal anti-phospho-p38 antibody (Cell Signaling). Total Sty1 levels were detected with rabbit polyclonal anti-Hog1 (y-215) antibody (Santa Cruz Biotechnology Inc). Mouse monoclonal anti-PSTAIR (anti-Cdc2, Sigma-Aldrich) was used for loading control. Immunoreactive bands were revealed with anti-rabbit or anti-mouse-HRP-conjugated secondary antibodies (Sigma-Aldrich) and the ECL system (GE-Healthcare). Densitometric quantification of Western blot signals as of 16-bit .jpg digital images of blots was performed using ImageJ [56]. Relative Units for Sty1 activation were estimated by determining the signal ratio of the anti-phospho-p38 blot (activated Sty1) with respect to the anti-Hog1 blot (total Sty1) at each time point. Relative Units for total Sty1 were calculated by determining the signal ratio of the anti-Hog1 blot (total Sty1) with respect to the anti-Cdc2 blot (loading control). Unless otherwise stated, results shown correspond to experiments performed as biological triplicates. Mean relative units ± SD and/or representative results are shown. *P*-values were analyzed by unpaired Student´s *t* test.

### Microscopy analysis

Fluorescence images were obtained with a Leica DM4000B microscope equipped with a Leica DC400F camera, and processed using IM500 Image Manager software. Calcofluor white and DAPI were employed, respectively, for cell wall/septum and nuclei staining as described [51]. To determine cell length at division the yeast strains were grown in YES medium to an A_600_ of 0.5 and stained with calcofluor white. A minimum of 200 septated cells were scored per triplicate for each strain. To quantify the increase in cell length during hyphal induction experiments with CPT, samples were taken at the indicated times and fixed immediately with formaldehyde [65]. After staining with calcofluor white and/or DAPI the length of mononuclear late G2 cells (n≥200) was measured. Three biological replicates were scored for each strain genotype.

### GC/MS analysis

Phenylethanol in the conditioned medium was extracted with ethyl acetate at a ratio of 5:1, dried with N_2_ at room temperature, and resuspended in dichloromethane. The samples were analyzed by a GC 7890A (Agilent Technologies, Santa Clara, CA, USA) coupled to a MS 5977 (Agilent Technologies, Santa Clara, CA, USA) with an inert EI source and a Quadrupole detector. The column was a 30m Agilent Technologies HP INNOWAX, 0.25 mm of internal diameter and 0.25 μM of flow with an operating range from 40 to 270°C. GC used a 1μl sample, injector and detector temperatures of 240 (splitless mode) and 150°C, respectively. The oven temperature program started at 120°C, increased at 3°C/min to reach 220°C, and then at 10°C/min to reach 250°C. The mass spectra were recorded in range of 35–300 m/z. Detection and quantification of phenylethanol was performed in the extracted-ion chromatogram (EIC) selecting a characteristic ion of m/z 91.

### HPLC/MS analysis

The detection and quantification of tyrosol and tryptophol in conditioned medium was determined with a HPLC/MS system consisting of a 1290 Infinity II Series HPLC (Agilent Technologies, Santa Clara, CA, USA), and connected to a 6550 Q-TOF Mass Spectrometer (Agilent Technologies, Santa Clara, CA, USA) using an Agilent Jet Stream Dual electrospray (AJS-Dual ESI) interface. Aromatic alcohols in 1 ml samples from conditioned medium were extracted with 3 ml of ethyl-acetate and vortexed for 1 min. After centrifuging for 5 min, the organic phase was transferred to a new tube, evaporated, and resuspended in 100 μl of MilliQ water. Aromatic alcohol standards were disolved in ethanol. Samples and standards (20 μl each) were injected into a Waters XBridge C18 HPLC column (2.1 × 100 mm, 5 μm, Agilent Technologies), thermostated at 30°C, and eluted at a flow rate of 400 μl/min. Mobile phase A (0.1% formic acid (w/v) in MilliQ water), and mobile phase B (0.1% formic acid (w/v) in acetonitrile), were used for the chromatographic separation. The initial HPLC running conditions were solvent A:B 95:5 (v/v). The gradient elution program was 5% solvent B for 3 min; a linear gradient from 5 to 100% solvent B in 10 min; 2 min at constant 100% solvent B. The mass spectrometer was operated in the positive mode. Profile data were acquired for both MS and MS/MS scans in extended dynamic range mode. MS and MS/MS mass range was 50–250 m/z and scan rates were 8 spectra/sec for MS and 3 spectra/sec for MS/MS. Tyrosol was detected as the 130.1591 m/z, whereas tryptophol was detected as the [M+H]+ ion at 162.0909 m/z, and confirmed with the transition 162.0909 > 144.0807.

### HPLC/UV analysis

Alternatively, concentrations of 2-phenylethanol, tyrosol and tryptophol in conditioned medium were determined using an Agilent 1100 Series high–performance liquid chromatography system (HPLC, Agilent Technologies, Santa Clara, CA, USA) equipped with a thermostated μ-wellplate autosampler, a quaternary pump, and a multiple wavenumber detector. Samples and standards (40 μl) were injected into a Zorbax Eclipse XDB-C18 HPLC column (Agilent Technologies), thermostated at 30°C, and eluted at a flow rate of 400 μl/min. Mobile phase A (0.1% acetic acid (w/v) in MilliQ water) and mobile phase B (0.1% formic acid (w/v) in methanol), were used for the chromatographic separation. The initial HPLC running conditions were solvent A:B 95:5 (v/v). The gradient elution program was 5% solvent B for 5 min; a linear gradient from 5 to 100% solvent B in 20 min; 5 min at constant 100% solvent B. The detection wavelength was 210 nm.

## Supporting information

S1 Fig Detection of phenylethanol and tryptophol in *S*. *japonicus* growth medium. (A) Detection of phenylethanol by GC/MS analysis. Upper panel shows a typical gas chromatogram of an extracted sample from conditioned medium. Lower panel shows the mass spectra correspondent to peak visible at a ~9.1 min retention time that is identified as phenylethanol by the presence of the characteristic ion of m/z 91. (B) Detection of tryptophol by HPLC/MS analysis. Upper panel shows a liquid chromatogram of an extracted sample from conditioned medium. Lower panel: tryptophol was detected as the peak with a ~4.3 min retention time by the characteristic ion of m/z 162.09.
(EPS)

S2 Fig Growth curves of *S*. *japonicus* wild type strain in YES medium (6% glucose) supplemented with the indicated amounts of phenylethanol, tryptophol and tyrosol. (EPS)

S3 Fig Conservation of Sty1 and Atf1 in *S*. *japonicus* and *S*. *pombe*. (A) ClustalW analysis of amino-acids sequences of Sty1 MAPKs in *S*. *pombe* and *S*. *japonicus*. The analysis was performed on the genome.jp server (https://www.genome.jp/tools-bin/clustalw) using the default settings. Identical amino-acids are marked with * and shaded in blue. Conserved residues/motifs involved in ATP binding are shaded in yellow. Putative MAPKK (-DXXD- motif) and common (-ED- motif) docking sites are shaded in purple. The conserved -TGY- activation loop specific of MAP kinases of the p38 type is shaded in green. (B) Anti-Hog1 and phospho-p38 antibodies specifically detect the respective total and dually phosphorylated isoforms of Sty1 in total extracts from *S*. *japonicus* wild type cells, but not in the *sty1Δ* mutant (C) Anti-Hog1 and phospho-p38 antibodies were employed to detect the respective total and dually phosphorylated isoforms of Sty1 in samples from wild type *S*. *japonicus* cells growing in YES medium at the indicated cellular densities. (D) ClustalW analysis of amino-acids sequences of Atf1 transcription factor in *S*. *pombe* and *S*. *japonicus*. Identical amino-acids are marked with * and shaded in blue. The conserved HRA (recombination), osmotic stress response, and basic-leucine zipper (DNA binding) domains are shown. Putative MAPK-dependent phoshorylation sites (SP/TP) present in both proteins are underlined.
(EPS)

S4 Fig Sty1 regulates chronological lifespan in *S*. *japonicus*. (A) Upper panel. *S japonicus* wild type and *sty1Δ* strains were grown in YES medium. Samples were taken at the indicated times (days), incubated with phloxine B, and the percentage of viable (non-stained) cells was determined microscopically. Results from an experiment performed per triplicate are shown. Lower panel. Serially diluted cells from samples described above were spotted on YES solid plates, incubated for 3 days at 30°C, and photographed. Results representative of three independent experiments are shown. (B) Serially diluted cells of wild type, *sty1*Δ, *atf1*Δ and *pyp1*Δ strains were spotted on YES plates supplemented with or without 0.2 μM CPT and incubated for 3 days at 30°C. Results representative of three independent experiments are shown.
(EPS)

S5 Fig Validation of mRNA levels of selected up- and down-regulated genes identified in RNAseq experiments. They were measured by qPCR from total RNA extracted from cell samples corresponding to *S*. *japonicus* wild type, *sty1*Δ, and *atf1*Δ strains growing exponentially in YES medium. Results are shown as relative fold expression (mean ± SD) from three biological repeats. *, *P*<0.05; **, *P*<0.005; ***, *P*<0.001, as calculated by unpaired Student´s *t* test.
(EPS)

S6 Fig Frequency of GO terms for common up-regulated (A) and down-regulated (B) genes in sty1Δ and atf1Δ mutants.
(EPS)

S7 Fig Conservation of Nrg1 in *S*. *japonicus* and *C*. *albicans*. ClustalW analysis of amino-acids sequences of Nrg1 transcription factor in *S*. *japonicus* and *C*. *albicans*. The analysis was performed on the genome.jp server (https://www.genome.jp/tools-bin/clustalw) using the default settings. Identical amino-acids are marked with * and shaded in blue. The putative C_2_H_2_ zinc finger region and the conserved cysteine and histidine residues are marked with a black line and shaded in red, respectively. The putative MAPK consensus phosphorylation sites are shown underlined.
(EPS)

S8 Fig Exogenously added farnesol is a QSM in *S*. *japonicus*. (A) Exponentially growing *S*. *japonicus* wild type cells were inoculated at an initial cell density of 10^6^ cells/ml in high glucose (6%) YES medium and incubated for 6h with 0.2 μM CPT without further treatment (untreated) or in the presence of the indicated amounts of farnesol. Cell length is represented as box and whisker plots. Data obtained after quantification of one experiment performed per triplicate (n≥ 200 cells/sample) is shown. ****, *P*<0.0001, as calculated by unpaired Student´s *t* test. (B) Cells from *S*. *japonicus* wild type cells growing in YES medium (2.10^6^) were spotted on YEMA plates in the absence or presence of 40 μM farnesol, incubated at 30°C for 7 days, and then photographed. The total area of mycelial expansion (expressed as relative units) was measured (n≥6) and is represented as scatter plot. **, *P*<0.005, as calculated by unpaired Student´s *t* test.
(EPS)

S1 Table List of up-regulated genes in exponentially growing *sty1*Δ cells. (PDF)

S2 Table List of down-regulated genes in exponentially growing *sty1*Δ cells. (PDF)

S3 Table List of up-regulated genes in exponentially growing *atf1*Δ cells. (PDF)

S4 Table List of down-regulated genes in exponentially growing *atf1*Δ cells. (PDF)

S5 Table Common up-regulated genes in *sty1*Δ and *atf1*Δ cells. (PDF)

S6 Table Common down-regulated genes in *sty1*Δ and *atf1*Δ cells. (PDF)

S7 Table Common up-regulated genes in *sty1*Δ cells and down-regulated in *atf1*Δ cells. (PDF)

S8 Table Common down-regulated genes in *sty1*Δ cells and up-regulated in *atf1*Δ cells. (PDF)

S9 Table Yeast strains used in this study. (PDF)

S10 Table Oligonucleotides used in this work. (PDF)
