# Correction: Quorum sensing and stress-activated MAPK signaling repress yeast to hypha transition in the fission yeast *Schizosaccharomyces japonicus*

**DOI:** 10.1371/journal.pgen.1008282

**Published:** 2019-07-19

**Authors:** Elisa Gómez-Gil, Alejandro Franco, Marisa Madrid, Beatriz Vázquez-Marín, Mariano Gacto, Jesualdo Fernández-Breis, Jero Vicente-Soler, Teresa Soto, José Cansado

The following information is missing from the Funding statement: This study was co-funded by European Regional Development Fund (FEDER).
