# Supplementary material for: Quorum sensing and stress-activated MAPK signaling repress yeast to hypha transition in the fission yeast Schizosaccharomyces japonicus
Source: PLoS Genet. 2019 May 31;15(5):e1008192. doi: 10.1371/journal.pgen.1008192 (PMC6561576; doi:10.1371/journal.pgen.1008192)
Supplement: S1 Table — (PDF) [file pgen.1008192.s009.pdf]

S1 Table. sty1Δ up-regulated genes

Gene	STY1_mean	CONTROL_me	log2FC	Description
SJAG_00006	2,828265	1,0102715	1,48517425	hypothetical protein
SJAG_00084	366,671	166,756	1,1367475	adenylyl-sulfate kinase
SJAG_00110	7,22275	2,445735	1,56228014	But2 family protein
SJAG_00124	1,66348	0,571147	1,54227051	transcriptional regulator NRG1
SJAG_00179	382,0535	20,78725	4,20000386	glutathione S-transferase Gst2
SJAG_00257	81,33485	26,4993	1,61791939	hypothetical protein
SJAG_00258	2,58856	1,168465	1,14753524	hypothetical protein
SJAG_00780	8,07228	3,74854	1,10664742	hypothetical protein
SJAG_00927	4,638925	1,62873	1,51004306	hypothetical protein
SJAG_01239	53,99655	25,8489	1,06276434	protein phosphatase Fmp31
SJAG_01416	102,9918	45,6613	1,17348564	pig-L
SJAG_01552	1,30329	0,262511	2,31170836	hypothetical protein
SJAG_01690	25,12765	2,77317	3,17966779	NADP-dependent L-serine/L-allo-threonine dehydrogenase ydfG
SJAG_01986	282,2025	31,3656	3,1694757	alcohol dehydrogenase
SJAG_02076	24,68605	10,296	1,26161205	hypothetical protein
SJAG_02106	32,4933	9,72536	1,74031871	hypothetical protein
SJAG_02125	1,43034	0,3757585	1,92848048	urea transporter
SJAG_02788	8,671825	3,759075	1,20595794	fungal protein
SJAG_02812	2112,84	992,902	1,08946028	translation elongation factor eIF5A
SJAG_02834	48,4188	10,446155	2,21259531	siderophore iron transporter 1
SJAG_02928	5,475095	2,62326	1,06152319	hypothetical protein
SJAG_02955	9,830835	2,923165	1,74978269	general amino acid permease AGP2
SJAG_03475	827,443	306,516	1,43269755	sulfate adenylyltransferase
SJAG_03492	152,82	28,58845	2,41832906	NADP-dependent L-serine/L-allo-threonine dehydrogenase ydfG
SJAG_03494	310,1275	154,196	1,00809612	glutamate-cysteine ligase regulatory subunit
SJAG_03643	39,9933	18,15125	1,13968942	arrestin Aly1
SJAG_03759	166,094	76,68555	1,1149733	phosphoglycerate mutase
SJAG_03820	1,533005	0,6743025	1,18489455	hexose transporter Ght8
SJAG_03821	62,91495	11,698135	2,42712433	hypothetical protein
SJAG_03822	2,992785	0,3968775	2,91472296	alcohol dehydrogenase Adh4
SJAG_03961	237,067	118,08965	1,00541233	5-aminolevulinate synthase
SJAG_04124	66,65625	33,2334	1,00410626	DUF1776 family protein

S1 Table. sty1Δ up-regulated genes

SJAG_04269	4,114675	1,878215	1,13141626	hypothetical protein
SJAG_04365	11,09255	5,15836	1,10460669	hypothetical protein
SJAG_04376	1,356375	0,239964	2,49886621	peptidase
SJAG_04743	338,644	168,974	1,00296816	ferric reductase transmembrane component
SJAG_04833	1,768965	0,5627125	1,65243559	hypothetical protein
SJAG_05015	173,3225	73,83375	1,23110661	NADPH dehydrogenase
SJAG_05173	1,091205	0,272538	2,00139286	hypothetical protein
SJAG_06097	95,57135	12,44505	2,94100617	hypothetical protein
SJAG_06596	58,5555	27,9081	1,06912078	hypothetical protein
SJAG_06627	3,01731	1,146061	1,39657909	hypothetical protein
SJAG_16028	34,78845	9,8846	1,81535391	n/a
SJAG_16042	36,4151	0,5	6,1864649	n/a
SJAG_16075	151,8425	45,91295	1,72560261	n/a
SJAG_16103	13,7006	0,5	4,77616717	n/a
SJAG_16118	68,82765	31,54525	1,12556547	n/a
SJAG_16119	7,6375	0,5	3,93310047	n/a
SJAG_16127	45,75075	16,9944	1,42873597	n/a
SJAG_16129	7,5881	0,5	3,92373869	n/a
SJAG_16303	10,0963	0,5	4,33575478	n/a
SJAG_16443	42,96995	12,0673	1,83222518	n/a
SJAG_16445	26,1039	12,46605	1,06626096	n/a
