# Supplementary material for: Quorum sensing and stress-activated MAPK signaling repress yeast to hypha transition in the fission yeast Schizosaccharomyces japonicus
Source: PLoS Genet. 2019 May 31;15(5):e1008192. doi: 10.1371/journal.pgen.1008192 (PMC6561576; doi:10.1371/journal.pgen.1008192)
Supplement: S2 Table — (PDF) [file pgen.1008192.s010.pdf]

S2 Table. sty1Δ down-regulated genes

Gene	STY1_mean	CONTROL_me	log2FC	Description
SJAG_00085	33,5832	93,55445	-1,47806657	DUF423 protein
SJAG_00097	0,8823105	3,21019	-1,86330033	5-aminolevulinate synthase
SJAG_00099	8,79049	24,04305	-1,45160443	Delta(12) fatty acid desaturase
SJAG_00138	8,03058	36,25915	-2,17476901	fungal protein
SJAG_00145	15,8531	44,5811	-1,49166723	RNA-binding protein
SJAG_00223	48,6741	929,2205	-4,25479477	hsp9-like protein
SJAG_00260	2,68744	7,657265	-1,51059664	succinate dehydrogenase iron-sulfur protein subunit
SJAG_00265	3,643795	10,015555	-1,45872867	D-arabinono-1,4-lactone oxidase
SJAG_00266	38,37745	95,6701	-1,31780925	transcription factor Atf1
SJAG_00372	156,1285	526,95	-1,75493216	plasma membrane proteolipid Pmp3
SJAG_00409	229,6135	468,7315	-1,02955428	glycerol-3-phosphate dehydrogenase Gpd1
SJAG_00449	8,891805	22,5877	-1,34498916	cytochrome C oxidase copper chaperone Cox17
SJAG_00452	29,10365	185,126	-2,66923553	ubiquitin
SJAG_00555	7,298095	16,41875	-1,16975246	zf-PARP type zinc finger protein
SJAG_00625	76,95695	214,6395	-1,47979207	hypothetical protein
SJAG_00635	7,916765	25,35125	-1,67907395	ornithine carbamoyltransferase Arg3
SJAG_00667	33,0519	81,79525	-1,30728386	endo-1,3-beta-glucanase Eng1
SJAG_00699	62,72575	145,7685	-1,21654927	tspO/peripheral benzodiazepine receptor
SJAG_00709	0,725534	1,94908	-1,42567818	hypothetical protein
SJAG_00788	1,18238	3,05638	-1,37013015	hypothetical protein
SJAG_00789	4,04648	64,51565	-3,9949098	hypothetical protein
SJAG_00812	90,649	187,0795	-1,04528847	phosphatidyl-N-methylethanolamine N-methyltransferase
SJAG_00979	0,559284	2,79279	-2,32005413	transcription factor atf31
SJAG_00980	0,481918	1,42571	-1,56482096	ATP-dependent DNA helicase Rdh54
SJAG_00981	14,18285	53,25545	-1,90878171	fungal cellulose binding domain-containing protein
SJAG_00993	15,34695	32,4833	-1,08174624	STE/STE7/MEK1 protein kinase Byr1
SJAG_01084	11,718	31,05845	-1,40625948	CAMK/CAMK1 protein kinase Srk1
SJAG_01427	8,280035	31,116	-1,90994784	alpha,alpha-trehalose-phosphate synthase
SJAG_01432	23,1352	46,4439	-1,00539955	hydroxyacid dehydrogenase
SJAG_01490	1,989295	6,141705	-1,62638198	ubiquinol-cytochrome-c reductase complex subunit 8
SJAG_01531	42,26305	105,29145	-1,3169195	alpha,alpha-trehalose-phosphate synthase
SJAG_01540	3,23956	6,63801	-1,03495292	Cullin 4

S2 Table. sty1Δ down-regulated genes

SJAG_01578	1,0064045	2,492915	-1,30862341	fungal protein
SJAG_01725	14,7266	57,46855	-1,96434826	transcription factor Atf21
SJAG_01757	0,463756	1,14554	-1,30458998	hypothetical protein
SJAG_01795	10,906255	25,53405	-1,22726659	hydrolase
SJAG_01815	10,44543	45,781	-2,13187709	hypothetical protein
SJAG_01869	15,6442	60,43445	-1,94974329	NADH/NADPH dependent indole-3-acetaldehyde reductase AKR3C2
SJAG_01905	2,58805	10,483995	-2,01825118	progesterone binding protein
SJAG_01968	128,5035	426,0035	-1,72905763	pepsin A
SJAG_02013	15,01195	52,2071	-1,79813463	tyrosine phosphatase Pyp1
SJAG_02122	1,80947	5,272385	-1,54288853	hypothetical protein
SJAG_02338	8,6195	24,4972	-1,50694077	non classical export pathway protein
SJAG_02432	85,245	214,9425	-1,33426365	vacuolar serine protease Isp6
SJAG_02442	11,92445	32,49385	-1,44624396	hypothetical protein
SJAG_02496	13,83025	52,88875	-1,93513364	D-amino acid oxidase
SJAG_02550	35,49925	71,71425	-1,01447127	DUF1941 family protein
SJAG_02569	2,09771	4,86485	-1,21358008	transcription factor
SJAG_02612	7,964325	18,86985	-1,24445896	fungal protein
SJAG_02626	88,75885	238,559	-1,42638323	protein kinase inhibitor
SJAG_02701	23,01955	50,6588	-1,13795327	bromodomain protein
SJAG_02744	15,508	85,72525	-2,46670757	cytochrome c
SJAG_02950	2,374475	7,339975	-1,62816658	galactokinase Gal1
SJAG_02951	2,412265	11,48828	-2,25170251	gal10
SJAG_02975	3,28861	7,20358	-1,13123614	hypoxia induced family protein
SJAG_02983	0,5	1,342175	-1,42457279	hypothetical protein
SJAG_02984	0,6448745	1,71266	-1,40914845	phosphoglycerate mutase family protein
SJAG_03063	47,9229	160,5175	-1,74394347	dienelactone hydrolase
SJAG_03201	2,70036	7,198125	-1,4144694	hypothetical protein
SJAG_03318	12,2909	24,67255	-1,00531627	N-acetyltransferase
SJAG_03388	18,3117	39,3405	-1,10324956	transcription factor Hsr1
SJAG_03603	38,1346	89,70465	-1,2340822	high-mobility group non-histone chromatin protein
SJAG_03606	4,75909	22,6118	-2,2483182	hexose transporter Ght6
SJAG_03786	426,936	1153,405	-1,43380746	aldehyde dehydrogenase
SJAG_03794	26,2432	52,5063	-1,00054689	DNAJ domain-containing protein Psi1

S2 Table. sty1Δ down-regulated genes

SJAG_03803	5,80574	13,00355	-1,16335367	hypothetical protein
SJAG_03804	8,008845	19,45315	-1,28033768	D-lactate dehydrogenase
SJAG_03818	0,588267	1,641725	-1,48066947	gal10
SJAG_03830	2,85949	18,212145	-2,67107109	hypothetical protein
SJAG_03958	7,90094	22,58115	-1,51502275	xylose and arabinose reductase
SJAG_04007	214,8525	774,7965	-1,85047087	fungal protein
SJAG_04008	7,278295	15,30665	-1,07248614	cytochrome c heme lyase
SJAG_04043	1,145435	16,41645	-3,84117469	hypothetical protein
SJAG_04055	132,8925	304,623	-1,19676519	heat shock protein S
SJAG_04135	1,690565	6,730355	-1,99317712	amino acid permease
SJAG_04227	9,251075	20,0122	-1,11318685	fungal protein
SJAG_04297	0,2512055	2,202005	-3,13187779	sulfonate dioxygenase
SJAG_04298	3,44859	21,84355	-2,66312882	hydantoin racemase family protein
SJAG_04299	0,190673	1,105964	-2,53613195	uricase
SJAG_04312	11,0821	25,6105	-1,20850413	AGC/PKA protein kinase Pka1
SJAG_04375	45,27445	156,9915	-1,79391743	septin Spn3
SJAG_04430	33,1786	70,6924	-1,09130211	hypothetical protein
SJAG_04458	2,66698	9,036025	-1,76048126	NAD binding dehydrogenase
SJAG_04568	1,154575	2,69024	-1,22037299	decaprenyl diphosphate synthase subunit Dps1
SJAG_04625	0,9118215	4,53031	-2,31278644	DUF1761 family protein
SJAG_04660	14,0903	29,39945	-1,06108684	xylose and arabinose reductase
SJAG_04662	1,698225	3,68487	-1,11758611	hypothetical protein
SJAG_04673	7,478895	21,97705	-1,55510071	thiamine transporter Thi9
SJAG_04682	8,80014	19,2508	-1,12932002	CCCH tandem zinc finger protein
SJAG_04711	18,9278	42,69435	-1,17353843	GTP cyclohydrolase II
SJAG_04713	12,0446	24,56965	-1,02849082	uracil phosphoribosyltransferase
SJAG_04789	1,192355	2,573135	-1,10971332	alpha-amylase Aah4
SJAG_04859	23,1768	63,57825	-1,45585192	alpha,alpha-trehalase Ntp1
SJAG_04868	1,2909	2,82928	-1,13205771	chitin synthase I
SJAG_05005	3,9484	13,89165	-1,81487791	fungal protein
SJAG_05181	24,6118	55,4105	-1,17080921	glutathione S-transferase Gst3
SJAG_05305	14,42907	53,44455	-1,88906452	membrane protein complex assembly protein
SJAG_05558	40,4894	91,0148	-1,1685569	fungal protein

S2 Table. sty1Δ down-regulated genes

SJAG_05896	1,846875	12,0208	-2,70237478	hypothetical protein
SJAG_06002	2,33124	9,252875	-1,98880416	hypothetical protein
SJAG_06111	0,357933	1,31765	-1,88020574	hypothetical protein
SJAG_16057	0,5	8,91508	-4,15624774	n/a
SJAG_16122	0,5	20,83475	-5,38091988	n/a
SJAG_16123	18,41485	37,4238	-1,02308641	n/a
SJAG_16142	0,5	19,4437	-5,28123087	n/a
SJAG_16183	11,58695	38,0743	-1,71631665	n/a
