# Supplementary material for: Quorum sensing and stress-activated MAPK signaling repress yeast to hypha transition in the fission yeast Schizosaccharomyces japonicus
Source: PLoS Genet. 2019 May 31;15(5):e1008192. doi: 10.1371/journal.pgen.1008192 (PMC6561576; doi:10.1371/journal.pgen.1008192)
Supplement: S3 Table — (PDF) [file pgen.1008192.s011.pdf]

S3 Table. atf1Δ up-regulated genes

Gene	ATF1_mean	CONTROL_me	log2FC	Description
SJAG_00006	3,52446	1,0102715	1,80265918	hypothetical protein
SJAG_00026	2,218455	0,5596825	1,98687475	hypothetical protein
SJAG_00075	39,1397	13,81595	1,50229793	trichothecene 3-O-acetyltransferase
SJAG_00084	479,4945	166,756	1,5237756	adenylyl-sulfate kinase
SJAG_00091	43,2244	15,7396	1,45744706	fungal protein
SJAG_00097	15,22735	3,21019	2,2459343	5-aminolevulinate synthase
SJAG_00099	58,62485	24,04305	1,2858924	Delta(12) fatty acid desaturase
SJAG_00110	10,45211	2,445735	2,09545421	But2 family protein
SJAG_00121	44,57215	2,62936	4,08335897	hypothetical protein
SJAG_00124	23,6943	0,571147	5,37453412	transcriptional regulator NRG1
SJAG_00144	32,15515	15,1846	1,08244091	GRIP domain-containing protein
SJAG_00179	127,9507	20,78725	2,62181722	glutathione S-transferase Gst2
SJAG_00191	17,8584	6,109495	1,54747779	hypothetical protein
SJAG_00211	20,36705	10,15739	1,00370729	DUF803 domain-containing protein
SJAG_00237	18,19995	8,80279	1,04790173	hexitol dehydrogenase
SJAG_00238	1,42617	0,354537	2,00813786	glutathione S-transferase Gst1
SJAG_00240	4,04428	1,453455	1,47639648	alcohol dehydrogenase Adh4
SJAG_00242	337,2605	51,89395	2,7002251	hypothetical protein
SJAG_00258	2,35646	1,168465	1,01200667	hypothetical protein
SJAG_00358	15,3797	5,249965	1,55064765	tRNA(5-methylaminomethyl-2-thiouridylate)-methyltransferase
SJAG_00415	105,15965	33,7224	1,64080212	hypothetical protein
SJAG_00451	318,584	147,304	1,11287721	carboxypeptidase
SJAG_00528	2,839715	0,2986125	3,24939968	hypothetical protein
SJAG_00556	20,5942	7,48616	1,4599403	hypothetical protein
SJAG_00567	31,69675	13,29445	1,25351083	dymeclin 1
SJAG_00587	35,00715	0,518409	6,07741504	sphingoid long-chain base transporter RSB1
SJAG_00589	1,3611	0,538239	1,33845423	hypothetical protein
SJAG_00658	1616,32	420,4315	1,94277018	hypothetical protein
SJAG_00720	47,77305	20,3411	1,23179929	COP9/signalosome complex subunit Csn5
SJAG_00780	7,74405	3,74854	1,04675947	hypothetical protein
SJAG_00781	2,9274	1,0203895	1,52049993	P-factor pheromone Map2
SJAG_00788	10,336065	3,05638	1,75779122	hypothetical protein

S3 Table. atf1Δ up-regulated genes

SJAG_00804	13,24095	6,042075	1,13189064	SNARE Sft1
SJAG_00832	12,10435	3,880455	1,64122788	sulfatase modifying factor 1
SJAG_00927	3,357835	1,62873	1,04378388	hypothetical protein
SJAG_00963	4,003585	1,770175	1,17740044	HAL protein kinase Ppk8
SJAG_00976	18,0149	8,44574	1,0928949	cytochrome c oxidase subunit IV
SJAG_01007	38,9311	13,6062	1,51665891	nramp family manganese ion transporter
SJAG_01078	77,5771	18,2457	2,08807434	zinc homeostasis factor 1
SJAG_01130	53,8363	26,20115	1,03894913	hypothetical protein
SJAG_01168	53,5055	21,7217	1,30055018	membrane transporter
SJAG_01239	208,762	25,8489	3,01368434	protein phosphatase Fmp31
SJAG_01316	18,80215	6,42103	1,550021	hypothetical protein
SJAG_01346	70,62135	35,3004	1,00041987	hypothetical protein
SJAG_01372	160,385	60,0997	1,41610953	porphobilinogen synthase Hem2
SJAG_01437	158,7445	73,07695	1,11921828	synaptotagmin family C2 domain-containing protein
SJAG_01490	16,9497	6,141705	1,46454862	ubiquinol-cytochrome-c reductase complex subunit 8
SJAG_01505	2,83172	0,962165	1,55732239	acetate transporter
SJAG_01552	1,376025	0,262511	2,3900569	hypothetical protein
SJAG_01553	64,24945	6,28722	3,35318995	hypothetical protein
SJAG_01555	2,385185	0,384876	2,63163555	hypothetical protein
SJAG_01690	15,0661	2,77317	2,44169805	NADP-dependent L-serine/L-allo-threonine dehydrogenase ydfG
SJAG_01794	47,39245	22,8938	1,0497003	hypothetical protein
SJAG_01835	644,992	269,1125	1,26107186	1,3-beta-glucanosyltransferase Gas2
SJAG_01888	21,40515	10,5975	1,01423398	glucan 1,3-beta-glucosidase Exg2
SJAG_01919	109,405	11,7641	3,21721582	RNA-binding protein M
SJAG_01922	284,574	130,6165	1,1234667	citrate synthase Cit1
SJAG_01932	3,582	1,2158425	1,55880898	hypothetical protein
SJAG_01970	142,649	50,10715	1,50938125	thiamine-repressible acid phosphatase pho4
SJAG_01971	3,678785	1,274135	1,52971122	DNA-3-methyladenine glycosylase Mag1
SJAG_01975	85,19675	22,74925	1,90497941	kinetochore protein fta5
SJAG_01986	126,09425	31,3656	2,00724742	alcohol dehydrogenase
SJAG_02011	16,23465	6,60893	1,29658766	hypothetical protein
SJAG_02091	34,1475	6,51127	2,39076909	phospholipase B Plb1
SJAG_02106	22,11515	9,72536	1,18521147	hypothetical protein

S3 Table. atf1Δ up-regulated genes

SJAG_02113	2,42219	0,136585	4,14844108	amino acid permease
SJAG_02125	1,5768	0,3757585	2,06912204	urea transporter
SJAG_02148	15,5967	7,53826	1,04893735	glucose-6-phosphate 1-dehydrogenase
SJAG_02338	431,606	24,4972	4,13902616	non classical export pathway protein
SJAG_02339	49,7367	10,262295	2,27695739	RecA family ATPase Rlp1
SJAG_02344	57,10755	16,59905	1,78258082	spermine family transporter
SJAG_02350	3,404115	1,411325	1,27022953	fungal protein
SJAG_02567	81,39255	28,11675	1,5334669	phosphoprotein phosphatase
SJAG_02569	18,01355	4,86485	1,8886153	transcription factor
SJAG_02580	106,1455	29,4589	1,84926774	phosphoprotein phosphatase
SJAG_02665	53,65455	19,815	1,43710755	Vac7
SJAG_02735	1,0490945	0,2359565	2,15255182	siderophore iron transporter 1
SJAG_02785	62,02285	8,203305	2,91852265	protein kinase activator
SJAG_02788	10,9714	3,759075	1,54529803	fungal protein
SJAG_02794	84,2098	22,7356	1,88903506	cytochrome b5 reductase
SJAG_02925	56,11355	22,93625	1,29071965	hypothetical protein
SJAG_02941	11,552705	0,02960005	8,60841536	amino acid permease
SJAG_02946	12,14245	0,3109585	5,28719368	amino acid permease
SJAG_02950	15,5452	7,339975	1,08262212	galactokinase Gal1
SJAG_02955	12,6681	2,923165	2,11559699	general amino acid permease AGP2
SJAG_02963	46,6704	13,6811	1,7703236	NADP-dependent alcohol dehydrogenase
SJAG_02968	19,67215	4,56034	2,10894135	iron/zinc ion transporter
SJAG_02970	4,09503	1,25628	1,70471597	hypothetical protein
SJAG_02976	38,91575	9,70592	2,00341729	hypothetical protein
SJAG_03019	14,7853	4,85297	1,60722367	hypothetical protein
SJAG_03074	38,8345	14,58115	1,41323438	hypothetical protein
SJAG_03114	57,35875	27,7081	1,0497058	F1-ATPase delta subunit
SJAG_03204	29,98145	12,5273	1,25899465	phospholipase
SJAG_03221	100,9289	31,6753	1,67190915	Swi5 protein
SJAG_03244	352,0545	163,8685	1,10326023	bcap family protein
SJAG_03245	808,426	392,6545	1,04185529	RING finger-like protein Ini1
SJAG_03296	6,169375	0,7552015	3,03019081	inner membrane protein
SJAG_03297	2,3213	0,2212995	3,39086089	ribosomal protein subunit L19

S3 Table. atf1Δ up-regulated genes

SJAG_03303	2875,905	1131,475	1,34581131	manganese superoxide dismutase
SJAG_03304	46,36	12,03685	1,94542267	hypothetical protein
SJAG_03305	63,31895	24,8974	1,34664224	glycerol-3-phosphate O-acyltransferase
SJAG_03340	30,89875	7,856265	1,97563298	adaptor protein Ste4
SJAG_03361	591,3645	175,7075	1,75087187	rho GDP dissociation inhibitor Rdi1
SJAG_03493	10,5544	1,552435	2,7652398	peroxin Pex28/29
SJAG_03608	859,105	133,9883	2,68072744	hexose transporter Ght5
SJAG_03624	30,841	11,7394	1,39349087	phosphoric ester hydrolase Ssu72
SJAG_03643	55,77625	18,15125	1,61958204	arrestin Aly1
SJAG_03646	36,4625	13,58765	1,42411752	glucan 1,3-beta-glucosidase Exg3
SJAG_03647	28,72215	10,6076	1,43706547	MBF transcription factor complex subunit Rep1
SJAG_03735	47,51005	20,5794	1,2070318	transcription factor Esc1
SJAG_03766	14,02355	5,99919	1,22501198	hypothetical protein
SJAG_03778	2,36076	0,3692305	2,67665775	hypothetical protein
SJAG_03818	35,52125	1,641725	4,43539796	gal10
SJAG_03820	3,693395	0,6743025	2,45347971	hexose transporter Ght8
SJAG_03822	1,61908	0,3968775	2,02840859	alcohol dehydrogenase Adh4
SJAG_03824	5,118845	0,09830715	5,70237816	alpha-glucosidase
SJAG_03827	76,7924	2,4037	4,99763478	tryptophan permease
SJAG_03828	38,6559	18,67975	1,04921348	glyceraldehyde-3-phosphate dehydrogenase Tdh1
SJAG_03911	18,29925	6,66472	1,45716835	transcription factor Rsv1
SJAG_04152	34,07395	14,22205	1,26053977	NADH dehydrogenase
SJAG_04167	5,043805	0,611357	3,04442551	P-type ATPase
SJAG_04187	399,352	112,76655	1,82432176	hsp104-like protein
SJAG_04301	3156,08	95,5734	5,04538082	invertase
SJAG_04323	66,26155	20,7473	1,67524835	cardiolipin-specific phospholipase
SJAG_04348	36,7969	7,769485	2,24369335	hypothetical protein
SJAG_04373	51,48345	21,83735	1,23731094	hypothetical protein
SJAG_04374	59,85985	20,67045	1,53401887	hypothetical protein
SJAG_04376	1,387495	0,239964	2,53159268	peptidase
SJAG_04458	27,16445	9,036025	1,58795967	NAD binding dehydrogenase
SJAG_04568	9,150215	2,69024	1,76607076	decaprenyl diphosphate synthase subunit Dps1
SJAG_04590	69,95405	23,3712	1,58167577	hypothetical protein

S3 Table. atf1Δ up-regulated genes

SJAG_04662	7,929175	3,68487	1,10555704	hypothetical protein
SJAG_04664	13,42665	4,49326	1,57926494	protein disulfide isomerase
SJAG_04673	55,69455	21,97705	1,34153841	thiamine transporter Thi9
SJAG_04723	35,76975	10,256815	1,80215723	cell agglutination protein mam3
SJAG_04828	47,03065	20,94525	1,16697817	membrane transporter
SJAG_04830	197,9915	74,5486	1,40918533	NiCoT heavy metal ion transporter Nic1
SJAG_04831	55,62585	4,39112	3,66309461	hypothetical protein
SJAG_04833	4,808525	0,5627125	3,0951245	hypothetical protein
SJAG_04866	2,70414	0,3138275	3,10712616	hypothetical protein
SJAG_04927	405,6065	178,4075	1,18490451	inosine-uridine preferring nucleoside hydrolase
SJAG_04950	5,219825	1,71713	1,60400217	tat binding protein 1(TBP-1)-interacting protein
SJAG_04953	8039,635	2814,545	1,51422829	cytosolic thioredoxin Trx1
SJAG_05004	9,29404	3,515715	1,40248773	fungal protein
SJAG_05006	13,08465	2,171105	2,59137393	homeobox transcription factor Phx1
SJAG_05015	162,507	73,83375	1,13814952	NADPH dehydrogenase
SJAG_05201	1,572565	0,420429	1,90318556	hypothetical protein
SJAG_05221	33,45985	8,449645	1,98546834	translation release factor
SJAG_05311	45,2658	22,3167	1,02029774	sphingosine hydroxylase
SJAG_05348	1229,97	514,669	1,25690633	oligosaccharyltransferase subunit Ost4
SJAG_05358	9,34962	2,22779	2,06929449	hypothetical protein
SJAG_05896	27,1796	12,0208	1,17699131	hypothetical protein
SJAG_06097	231,7035	12,44505	4,21863591	hypothetical protein
SJAG_06585	30,83505	5,6095	2,4586271	hypothetical protein
SJAG_06627	5,154365	1,146061	2,16911087	hypothetical protein
SJAG_06641	6,19631	2,061835	1,58748044	hypothetical protein
SJAG_16081	91,40795	29,46455	1,63333941	n/a
SJAG_16103	18,42235	0,5	5,2033852	n/a
SJAG_16119	19,09405	0,5	5,25505124	n/a
SJAG_16129	13,5228	0,5	4,757322	n/a
SJAG_16142	49,0638	19,4437	1,3353562	n/a
SJAG_16303	8,32705	0,5	4,05780549	n/a
SJAG_16445	47,03775	12,46605	1,91581465	n/a
SJAG_16452	1224,585	37,1451	5,0429772	hypothetical protein
