# Supplementary material for: Quorum sensing and stress-activated MAPK signaling repress yeast to hypha transition in the fission yeast Schizosaccharomyces japonicus
Source: PLoS Genet. 2019 May 31;15(5):e1008192. doi: 10.1371/journal.pgen.1008192 (PMC6561576; doi:10.1371/journal.pgen.1008192)
Supplement: S4 Table — (PDF) [file pgen.1008192.s012.pdf]

S4 Table. atf1Δ down-regulated genes

Gene	ATF1_mean	CONTROL_me	log2FC	description
SJAG_00057	223,016	481,0225	-1,10895716	hexose transporter Ght2
SJAG_00085	16,69245	93,55445	-2,48661056	DUF423 protein
SJAG_00145	13,8455	44,5811	-1,68701506	RNA-binding protein
SJAG_00223	93,0847	929,2205	-3,31940502	hsp9-like protein
SJAG_00254	18,9159	39,04945	-1,04570281	aspartic proteinase sxa1
SJAG_00372	149,103	526,95	-1,82135679	plasma membrane proteolipid Pmp3
SJAG_00501	23,31405	95,9914	-2,04170551	ribulose phosphate 3-epimerase
SJAG_00625	101,89775	214,6395	-1,0747934	hypothetical protein
SJAG_00635	8,483495	25,35125	-1,57932624	ornithine carbamoyltransferase Arg3
SJAG_00668	11,3639	104,869	-3,20605832	superoxide dismutase Sod1
SJAG_00699	70,76145	145,7685	-1,04264348	tspO/peripheral benzodiazepine receptor
SJAG_00709	0,483755	1,94908	-2,01044483	hypothetical protein
SJAG_00789	16,67275	64,51565	-1,95215709	hypothetical protein
SJAG_00812	82,98055	187,0795	-1,17280635	phosphatidyl-N-methylethanolamine N-methyltransferase
SJAG_00890	19,92625	75,0591	-1,91335676	mannose-1-phosphate guanyltransferase
SJAG_00926	28,0165	62,37485	-1,1546877	transcription factor
SJAG_00970	19,0124	79,06055	-2,05601733	actin cortical patch component Lsb4
SJAG_00979	0,4138285	2,79279	-2,75460219	transcription factor atf31
SJAG_00980	0,258785	1,42571	-2,46185466	ATP-dependent DNA helicase Rdh54
SJAG_00981	0,652187	53,25545	-6,35149968	fungal cellulose binding domain-containing protein
SJAG_01017	10,16585	50,22795	-2,30475955	endo-1,3-beta-glucanase Eng2
SJAG_01057	13,4347	50,34585	-1,90590876	dihydroceramide delta-4 desaturase
SJAG_01066	14,25145	28,9241	-1,02116336	hypothetical protein
SJAG_01077	7,90111	17,50955	-1,14801475	cytochrome c oxidase subunit V
SJAG_01089	201,241	433,2365	-1,10623053	UTP-glucose-1-phosphate uridylyltransferase
SJAG_01154	0,9317715	1,903405	-1,03053446	phosphoprotein phosphatase
SJAG_01171	11,18231	33,86205	-1,59845107	glucan 1,3-beta-glucosidase
SJAG_01174	18,0904	49,8082	-1,46115897	Sad1 interacting factor 3
SJAG_01227	21,9884	63,47415	-1,52942654	glycosylceramide biosynthesis protein
SJAG_01262	7,85122	18,19225	-1,21233523	endonuclease Uve1
SJAG_01432	16,8482	46,4439	-1,46289466	hydroxyacid dehydrogenase
SJAG_01493	21,77135	89,5284	-2,03991453	copper transporter complex subunit Ctr5

S4 Table. atf1Δ down-regulated genes

SJAG_01725	10,944945	57,46855	-2,39250794	transcription factor Atf21
SJAG_01757	0,08403505	1,14554	-3,76889284	hypothetical protein
SJAG_01815	10,7115	45,781	-2,09558845	hypothetical protein
SJAG_01904	84,5536	211,904	-1,32547273	hypothetical protein
SJAG_01905	0,939823	10,483995	-3,47965569	progesterone binding protein
SJAG_01965	29,7269	117,3225	-1,98063879	hypothetical protein
SJAG_01968	146,617	426,0035	-1,53881289	pepsin A
SJAG_01982	19,79725	62,29695	-1,65386149	hypothetical protein
SJAG_01985	31,7183	75,83865	-1,25761783	hypothetical protein
SJAG_02016	20,90685	48,03675	-1,20016284	apoptosis-inducing factor Aif1
SJAG_02040	39,53965	107,375	-1,44128613	tubulin specific chaperone cofactor B
SJAG_02150	58,4723	159,739	-1,44989134	amino acid permease inda1
SJAG_02154	6,11194	37,79945	-2,62866296	GFO/IDH/MocA family oxidoreductase
SJAG_02191	67,21825	200,6245	-1,57757291	GFO/IDH/MocA family oxidoreductase
SJAG_02301	44,35205	89,6165	-1,01476359	ER associated protein disulfide isomerase Pdi2
SJAG_02442	11,63375	32,49385	-1,48185048	hypothetical protein
SJAG_02646	7,89437	20,0922	-1,3477395	cytochrome c1 Cyt1
SJAG_02696	9,059455	28,42915	-1,6498748	glucose-6-phosphate 1-dehydrogenase
SJAG_02697	361,251	2609,39	-2,8526391	ubiquitinated histone-like protein Uhp1
SJAG_02744	27,8429	85,72525	-1,62241072	cytochrome c
SJAG_02799	18,53065	40,6513	-1,133388	glucan 1,4-alpha-glucosidase
SJAG_02834	0,2257115	10,446155	-5,53234828	siderophore iron transporter 1
SJAG_02958	0,297207	1,1395535	-1,93892866	hexose transporter Ght5
SJAG_02983	0,5	1,342175	-1,42457279	hypothetical protein
SJAG_02987	384,3105	827,8625	-1,10711878	hypothetical protein
SJAG_03013	2,75083	7,06544	-1,36091242	chitin deacetylase Cda1
SJAG_03049	366,742	2338,635	-2,67282931	fungal protein
SJAG_03063	6,24407	160,5175	-4,68410007	dienelactone hydrolase
SJAG_03178	232,842	547,0645	-1,23235962	hypothetical protein
SJAG_03202	3,71334	10,3474	-1,47847898	hypothetical protein
SJAG_03266	355,161	874,8685	-1,30059302	NADP-specific glutamate dehydrogenase Gdh1
SJAG_03293	64,11735	281,5225	-2,13446353	succinate-semialdehyde dehydrogenase
SJAG_03363	14,1678	37,8714	-1,418493	leptomycin efflux transporter Pmd1

S4 Table. atf1Δ down-regulated genes

SJAG_03434	51,9867	159,035	-1,61312982	4-aminobutyrate aminotransferase
SJAG_03497	7,205865	14,45005	-1,00383096	ATP-binding cassette-type vacuolar membrane transporter Hmt1
SJAG_03784	457,4635	917,289	-1,0037197	hypothetical protein
SJAG_03794	21,30585	52,5063	-1,30124093	DNAJ domain-containing protein Psi1
SJAG_03815	30,0942	82,1334	-1,44848356	hsp16-like protein
SJAG_04031	0,933131	1,88392	-1,01358617	ferrichrome synthetase Sib1
SJAG_04043	0,40353	16,41645	-5,34632244	hypothetical protein
SJAG_04247	10,14334	29,0013	-1,51558479	hypothetical protein
SJAG_04269	0,8054355	1,878215	-1,22152125	hypothetical protein
SJAG_04297	1,0731775	2,202005	-1,03692903	sulfonate dioxygenase
SJAG_04375	73,8523	156,9915	-1,08797169	septin Spn3
SJAG_04430	13,7788	70,6924	-2,35910487	hypothetical protein
SJAG_04607	11,54383	24,6897	-1,09678735	CCAAT-binding factor complex subunit Php4
SJAG_04608	12,31475	53,01955	-2,10613709	flavin dependent monooxygenase
SJAG_04625	1,90336	4,53031	-1,25106132	DUF1761 family protein
SJAG_04637	17,13685	92,28685	-2,42902315	general amino acid permease GAP1
SJAG_04675	15,9272	177,694	-3,4798304	iron permease Fip1
SJAG_04743	5,903495	168,974	-4,83908815	ferric reductase transmembrane component
SJAG_04751	5,59802	13,15665	-1,23280364	ELLA family acetyltransferase
SJAG_04777	5,95359	12,8742	-1,11265101	STE/STE11 protein kinase
SJAG_04867	12,4708	25,47315	-1,03042336	ferrous iron transporter Pcl1
SJAG_04868	1,302775	2,82928	-1,11884702	chitin synthase I
SJAG_04944	34,83685	89,6238	-1,36326772	coproporphyrinogen III oxidase
SJAG_05005	6,91778	13,89165	-1,00583693	fungal protein
SJAG_05021	57,8696	171,5655	-1,56788189	RNA-binding protein Vip1
SJAG_05182	8,628025	17,9432	-1,05633494	allantoate permease
SJAG_05208	56,10865	113,516	-1,01660055	splicing factor 3B
SJAG_05213	11,95025	23,9631	-1,00377376	ATP-binding cassette transporter abc1
SJAG_05305	9,796965	53,44455	-2,44763605	membrane protein complex assembly protein
SJAG_05558	44,85575	91,0148	-1,02080823	fungal protein
SJAG_05889	12,9199	29,58455	-1,19524905	hypothetical protein
SJAG_06002	1,79372	9,252875	-2,366947	hypothetical protein
SJAG_16057	0,5	8,91508	-4,15624774	n/a

S4 Table. atf1Δ down-regulated genes

SJAG_16075	22,255265	45,91295	-1,04475445	n/a
SJAG_16118	0,5	31,54525	-5,97935088	n/a
SJAG_16122	0,5	20,83475	-5,38091988	n/a
SJAG_16183	17,3323	38,0743	-1,1353544	n/a
