# Supplementary material for: Quorum sensing and stress-activated MAPK signaling repress yeast to hypha transition in the fission yeast Schizosaccharomyces japonicus
Source: PLoS Genet. 2019 May 31;15(5):e1008192. doi: 10.1371/journal.pgen.1008192 (PMC6561576; doi:10.1371/journal.pgen.1008192)
Supplement: S5 Table — (PDF) [file pgen.1008192.s013.pdf]

S5 Table. Common up-regulated genes in sty1Δ and atf1Δ cells

**Gene**	**ATF1_mean**	**STY1_mean**	**CONTROL_me**	**log2FC_ATF1**	**log2FC_STY1**	Description
SJAG_00006	3,52446	2,828265	1,0102715	1,80265918	1,48517425	hypothetical protein
SJAG_00084	479,4945	366,671	166,756	1,5237756	1,1367475	adenylyl-sulfate kinase
SJAG_00110	10,45211	7,22275	2,445735	2,09545421	1,56228014	But2 family protein
SJAG_00124	23,6943	1,66348	0,571147	5,37453412	1,54227051	transcriptional regulator NRG1
SJAG_00179	127,9507	382,0535	20,78725	2,62181722	4,20000386	glutathione S-transferase Gst2
SJAG_00258	2,35646	2,58856	1,168465	1,01200667	1,14753524	hypothetical protein
SJAG_00780	7,74405	8,07228	3,74854	1,04675947	1,10664742	hypothetical protein
SJAG_00927	3,357835	4,638925	1,62873	1,04378388	1,51004306	hypothetical protein
SJAG_01239	208,762	53,99655	25,8489	3,01368434	1,06276434	protein phosphatase Fmp31
SJAG_01552	1,376025	1,30329	0,262511	2,3900569	2,31170836	hypothetical protein
SJAG_01690	15,0661	25,12765	2,77317	2,44169805	3,17966779	NADP-dependent L-serine/L-allo-threonine dehydrogenase ydfG
SJAG_01986	126,09425	282,2025	31,3656	2,00724742	3,1694757	alcohol dehydrogenase
SJAG_02106	22,11515	32,4933	9,72536	1,18521147	1,74031871	hypothetical protein
SJAG_02125	1,5768	1,43034	0,3757585	2,06912204	1,92848048	urea transporter
SJAG_02788	10,9714	8,671825	3,759075	1,54529803	1,20595794	fungal protein
SJAG_02955	12,6681	9,830835	2,923165	2,11559699	1,74978269	general amino acid permease AGP2
SJAG_03643	55,77625	39,9933	18,15125	1,61958204	1,13968942	arrestin Aly1
SJAG_03820	3,693395	1,533005	0,6743025	2,45347971	1,18489455	hexose transporter Ght8
SJAG_03822	1,61908	2,992785	0,3968775	2,02840859	2,91472296	alcohol dehydrogenase Adh4
SJAG_04376	1,387495	1,356375	0,239964	2,53159268	2,49886621	peptidase
SJAG_04833	4,808525	1,768965	0,5627125	3,0951245	1,65243559	hypothetical protein
SJAG_05015	162,507	173,3225	73,83375	1,13814952	1,23110661	NADPH dehydrogenase
SJAG_06097	231,7035	95,57135	12,44505	4,21863591	2,94100617	hypothetical protein
SJAG_06627	5,154365	3,01731	1,146061	2,16911087	1,39657909	hypothetical protein
SJAG_16103	18,42235	13,7006	0,5	5,2033852	4,77616717	n/a
SJAG_16119	19,09405	7,6375	0,5	5,25505124	3,93310047	n/a
SJAG_16129	13,5228	7,5881	0,5	4,757322	3,92373869	n/a
SJAG_16303	8,32705	10,0963	0,5	4,05780549	4,33575478	n/a
SJAG_16445	47,03775	26,1039	12,46605	1,91581465	1,06626096	n/a
