# Supplementary material for: Quorum sensing and stress-activated MAPK signaling repress yeast to hypha transition in the fission yeast Schizosaccharomyces japonicus
Source: PLoS Genet. 2019 May 31;15(5):e1008192. doi: 10.1371/journal.pgen.1008192 (PMC6561576; doi:10.1371/journal.pgen.1008192)
Supplement: S6 Table — (PDF) [file pgen.1008192.s014.pdf]

S6 Table. Common down-regulated genes in sty1Δ and atf1Δ cells

Gene	ATF1_mean	STY1_mean	CONTROL_me	log2FC_ATF1	log2FC_STY1	Description
SJAG_00085	16,69245	33,5832	93,55445	-2,48661056	-1,47806657	DUF423 protein
SJAG_00145	13,8455	15,8531	44,5811	-1,68701506	-1,49166723	RNA-binding protein
SJAG_00223	93,0847	48,6741	929,2205	-3,31940502	-4,25479477	hsp9-like protein
SJAG_00372	149,103	156,1285	526,95	-1,82135679	-1,75493216	plasma membrane proteolipid Pmp3
SJAG_00625	101,89775	76,95695	214,6395	-1,0747934	-1,47979207	hypothetical protein
SJAG_00635	8,483495	7,916765	25,35125	-1,57932624	-1,67907395	ornithine carbamoyltransferase Arg3
SJAG_00699	70,76145	62,72575	145,7685	-1,04264348	-1,21654927	tspO/peripheral benzodiazepine receptor
SJAG_00709	0,483755	0,725534	1,94908	-2,01044483	-1,42567818	hypothetical protein
SJAG_00789	16,67275	4,04648	64,51565	-1,95215709	-3,9949098	hypothetical protein
SJAG_00812	82,98055	90,649	187,0795	-1,17280635	-1,04528847	phosphatidyl-N-methylethanolamine N-methyltransferase
SJAG_00979	0,4138285	0,559284	2,79279	-2,75460219	-2,32005413	transcription factor atf31
SJAG_00980	0,258785	0,481918	1,42571	-2,46185466	-1,56482096	ATP-dependent DNA helicase Rdh54
SJAG_00981	0,652187	14,18285	53,25545	-6,35149968	-1,90878171	fungal cellulose binding domain-containing protein
SJAG_01432	16,8482	23,1352	46,4439	-1,46289466	-1,00539955	hydroxyacid dehydrogenase
SJAG_01725	10,944945	14,7266	57,46855	-2,39250794	-1,96434826	transcription factor Atf21
SJAG_01757	0,08403505	0,463756	1,14554	-3,76889284	-1,30458998	hypothetical protein
SJAG_01815	10,7115	10,44543	45,781	-2,09558845	-2,13187709	hypothetical protein
SJAG_01905	0,939823	2,58805	10,483995	-3,47965569	-2,01825118	progesterone binding protein
SJAG_01968	146,617	128,5035	426,0035	-1,53881289	-1,72905763	pepsin A
SJAG_02442	11,63375	11,92445	32,49385	-1,48185048	-1,44624396	hypothetical protein
SJAG_02744	27,8429	15,508	85,72525	-1,62241072	-2,46670757	cytochrome c
SJAG_02983	0,5	0,5	1,342175	-1,42457279	-1,42457279	hypothetical protein
SJAG_03063	6,24407	47,9229	160,5175	-4,68410007	-1,74394347	dienelactone hydrolase
SJAG_03794	21,30585	26,2432	52,5063	-1,30124093	-1,00054689	DNAJ domain-containing protein Psi1
SJAG_04043	0,40353	1,145435	16,41645	-5,34632244	-3,84117469	hypothetical protein
SJAG_04297	1,0731775	0,2512055	2,202005	-1,03692903	-3,13187779	sulfonate dioxygenase
SJAG_04375	73,8523	45,27445	156,9915	-1,08797169	-1,79391743	septin Spn3
SJAG_04430	13,7788	33,1786	70,6924	-2,35910487	-1,09130211	hypothetical protein
SJAG_04625	1,90336	0,9118215	4,53031	-1,25106132	-2,31278644	DUF1761 family protein
SJAG_04868	1,302775	1,2909	2,82928	-1,11884702	-1,13205771	chitin synthase I
SJAG_05005	6,91778	3,9484	13,89165	-1,00583693	-1,81487791	fungal protein
SJAG_05305	9,796965	14,42907	53,44455	-2,44763605	-1,88906452	membrane protein complex assembly protein

S6 Table. Common down-regulated genes in sty1Δ and atf1Δ cells

SJAG_05558	44,85575	40,4894	91,0148	-1,02080823	-1,1685569	fungal protein
SJAG_06002	1,79372	2,33124	9,252875	-2,366947	-1,98880416	hypothetical protein
SJAG_16057	0,5	0,5	8,91508	-4,15624774	-4,15624774	n/a
SJAG_16122	0,5	0,5	20,83475	-5,38091988	-5,38091988	n/a
SJAG_16183	17,3323	11,58695	38,0743	-1,1353544	-1,71631665	n/a
