# Supplementary material for: Quorum sensing and stress-activated MAPK signaling repress yeast to hypha transition in the fission yeast Schizosaccharomyces japonicus
Source: PLoS Genet. 2019 May 31;15(5):e1008192. doi: 10.1371/journal.pgen.1008192 (PMC6561576; doi:10.1371/journal.pgen.1008192)
Supplement: S7 Table — (PDF) [file pgen.1008192.s015.pdf]

S7 Table. Common genes up-regulated in sty1Δ cells and down-regulated in atf1Δ cells

**Gene**
SJAG_02834
SJAG_04269
SJAG_04743
SJAG_16075
SJAG_16118
