# Supplementary material for: Quorum sensing and stress-activated MAPK signaling repress yeast to hypha transition in the fission yeast Schizosaccharomyces japonicus
Source: PLoS Genet. 2019 May 31;15(5):e1008192. doi: 10.1371/journal.pgen.1008192 (PMC6561576; doi:10.1371/journal.pgen.1008192)
Supplement: S8 Table — (PDF) [file pgen.1008192.s016.pdf]

**S8 Table. Common genes up-regulated in sty1Δ cells and down-regulated in atf1Δ cells**

| Gene |
|---|
| SJAG_00097 |
| SJAG_00099 |
| SJAG_00788 |
| SJAG_01490 |
| SJAG_02338 |
| SJAG_02569 |
| SJAG_02950 |
| SJAG_03818 |
| SJAG_04458 |
| SJAG_04568 |
| SJAG_04662 |
| SJAG_04673 |
| SJAG_05896 |
| SJAG_16142 |
