# Supplementary material for: Quorum sensing and stress-activated MAPK signaling repress yeast to hypha transition in the fission yeast Schizosaccharomyces japonicus
Source: PLoS Genet. 2019 May 31;15(5):e1008192. doi: 10.1371/journal.pgen.1008192 (PMC6561576; doi:10.1371/journal.pgen.1008192)
Supplement: S9 Table — (PDF) [file pgen.1008192.s017.pdf]

S9 Table. ***S. japonicus* and *S. pombe* strains used in this work.**

S. japonicus strains	**Genotype**	**Source**
NIG2017	*h*$^{+}$	Furuya & Niki 2009
NIG5091	*h*$^{-}$ *ura4-D3*	Furuya & Niki 2009
NIG2028	*h-*	Furuya & Niki 2009
TSJ101	*h*$^{-}$ *ura4-D3 sty1::ura4*$^{+}$	This work
TSJ105	*h*$^{-}$ *ura4-D3 atf1::ura4*$^{+}$	This work
TSJ106	*h*$^{-}$ *ura4-D3 nrg1::ura4*$^{+}$	This work
TSJ108	*h*$^{-}$ *ura4-D3 nrg1::ura4*$^{+}$ *sty1::NatMX6*	This work
TSJ109	*h*$^{-}$ *ura4-D3 pyp1::ura4*$^{+}$	This work
TSJ110	*h*$^{-}$ *ura4-D3 pka1::ura4*$^{+}$	This work
S. pombe strains	**Genotype**	**Source**
L972	*h- prototroph*	U. Leupold
