# Supplementary material for: Quorum sensing and stress-activated MAPK signaling repress yeast to hypha transition in the fission yeast Schizosaccharomyces japonicus
Source: PLoS Genet. 2019 May 31;15(5):e1008192. doi: 10.1371/journal.pgen.1008192 (PMC6561576; doi:10.1371/journal.pgen.1008192)
Supplement: S10 Table — (PDF) [file pgen.1008192.s018.pdf]

**S10 Table. Oligonucleotides used in this work.**

| OLIGONUCLEOTIDE | SEQUENCE 5'-3' | Use |
|---|---|---|
| Leu1 FWD | GATGTCGGCGATGTGAATAAA | q-PCR |
| Leu1 REV | GGGAGGACGACAATCTTCTTA | q-PCR |
| Nrg1 (SJAG_00124) FWD | TAAGAACCAGAAGCCCAACT | q-PCR |
| Nrg1 (SJAG_00124) REV | AGGAGGACAATAGGGATGAAAT | q-PCR |
| Gst2 (SJAG_00179) FWD | CCTAACCCTTGGAAGGTAGTT | q-PCR |
| Gst2 (SJAG_00179) REV | TGTTAGCGTGGTCCACTAAT | q-PCR |
| Hsp9 (SJAG_00223) FWD | AGTATGTCACTCCCGACTCCT | q-PCR |
| Hsp9 (SJAG_00223) REV | TCTGCTCGTCGCTCTTGAAA | q-PCR |
| Atf31 (SJAG_00979) FWD | TTGAACCCACAAGCAGAAAG | q-PCR |
| Atf31 (SJAG_00979) REV | TCTGGCTTTAGAATGGGAAGA | q-PCR |
| SJAG_00981 FWD | CTTCCGGTTCCGAGTGTATAA | q-PCR |
| SJAG_00981 REV | CCTTCGGTACAACAAGTAGGA | q-PCR |
| SJAG_02834 FWD | GGTCACATAGCCAACACATAC | q-PCR |
| SJAG_02834 REV | TGTACCACGATAGCGAATCA | q-PCR |
| | | |
| URA4-JP-COMP-R | CTTCTTGGCGACTGCATTAGGATGC | Common oligonucleotide for confirmation of *ura4*$^+$ deletions. |
| NAT-COMP-R | CTCATGTAGAGCGCCTGCCGC | Common oligonucleotide for confirmation of *NatR* deletions. |
| NRG1Djp-W2 | AACCTAAACCTGACCGCAAAC | *nrg1*$^+$ deletion |
| NRG1Djp-X (URA4) | GAGCGGAAGAACGGAATCGTGGCGGCCCGGTGCAGTCGCTTTAGC AGGGTGC | *nrg1*$^+$ deletion (*ura4*$^+$) |
| NRG1Djp-Y (URA4) | GCAGTGCGGTATCGTATAATTAGTGTCCATAACTCTCGCTCGAGAGC CGCG | *nrg1*$^+$ deletion (*ura4*$^+$) |
| NRG1Djp-Z2 | TAGGAACAGATGAACTTGGCTCT | *nrg1*$^+$ deletion |
| NRG1Djp-COMP5' | CACTGCCTGTCTGCACGACAACCTG | Confirmation of *nrg1*$^+$ deletion. |
| STY1Djp-W | GCATGGGCGCGTTCGGTCTTGTATG | *sty1*$^+$ deletion |
| STY1Djp-X (URA4) | GAGCGGAAGAACGGAATCGTGGCGGCCACACCAGCACTGTGGACG TACTTC | *sty1*$^+$ deletion (*ura4*$^+$) |
| STY1Djp-Y (URA4) | GCAGTGCGGTATCGTATAATTAGTGTGACGATCTGCAGCAAGAATAC ATTG | *sty1*$^+$ deletion (*ura4*$^+$) |
| STY1Djp-Z | TGCTTGCACCTCACTATCCACATTATG | *sty1*$^+$ deletion |
| STY1Djp-X (NAT) | TTAATTAACCCGGGGATCCGACACCAGCACTGTGGACGTACTTC | *sty1*$^+$ deletion (*NatR*) |
| STY1Djp-Y (NAT) | GTTTAAACGAGCTCGAATTCGACGATCTGCAGCAAGAATACATTG | *sty1*$^+$ deletion (*NatR*) |
| STY1Djp-COMP5' | ATGGCTGAATTTGTTCGTACACAGAT | Confirmation of *sty1*$^+$ deletion. |
| ATF1Djp-W | TGGTGAACCACGGTTTGATTACC | *atf1*$^+$ deletion |
| ATF1Djp-X (URA4) | GAGCGGAAGAAC GGAATCGTGGCGGCCCGTTTGCTGAGGCAGAGGCTTC | *atf1*$^+$ deletion (*ura4*$^+$) |
| ATF1Djp-Y (URA4) | GCAGTGCGGTATCGTATAATTAGTGTCAATGACAAATCGTGATGCGC AG | *atf1*$^+$ deletion (*ura4*$^+$) |
| ATF1Djp-Z | TGAATGGCCTGCACTAAAGTGACG | *atf1*$^+$ deletion |
| ATF1Djp-COMP5' | GCTCTGCCCGATTCGCCTCTTACG | Confirmation of *atf1*$^+$ deletion. |
| PYP1Djp-W | CACGTGGAAACAGCTCGTATCTC | *pyp1*$^+$ deletion |
| PYP1Djp-X | GAGCGGAAGAACGGAATCGTGGCGGCCCATCATCGCGTTAAAGTGT CCAGG | *pyp1*$^+$ deletion (*ura4*$^+$) |
| PYP1Djp-Y | GCAGTGCGGTATCGTATAATTAGTGT*GA*CTTTGTCAGGATGATTCTC TCG | *pyp1*$^+$ deletion (*ura4*$^+$) |
| PYP1Djp-Z | AACGAAAGACGCACAAGTCACTGC | *pyp1*$^+$ deletion |
| PYPDjp-COMP5´ | GGCCTATTTAAAGGTAGTCTACCACC | Confirmation of *pyp1*$^+$ deletion. |
| PKA1Djp-W | TGTTTAGTGCGTAGAGGAACTCAGAG | *pka1*$^+$ deletion |

| | | |
|---|---|---|
| PKA1Djp-X (URA4) | GAGCGGAAGAACGGAATCGTGGCGGCC*CTCCGTATCGGTTCGATTTGGAAC* | *pka1*$^+$ deletion (*ura4*$^+$) |
| PKA1Djp-Y (URA4) | GCAGTGCGGTATCGTATAATTAGTGTTATCAACTGGGAATCTATCCTTAC | *pka1*$^+$ deletion (*ura4*$^+$) |
| PKA1Djp-Z | AATGTACCCGCTATCTCAAACGCTGC | *pka1*$^+$ deletion |
| PKA1Djp-COMP5’ | CTGCTGCATGTATGACGTTTGAGAAC | Confirmation of *pka1*$^+$ deletion. |
